# Insights into the synthesis and structural properties of pro-chiral 2-acetyl-*N*-aryl-2-(prop-2-yn-1-yl)pent-4-ynamides/-2-allyl-4-enamide derivatives through kinetics and energy frameworks†

**DOI:** 10.1039/d5ra02166a

**Published:** 2025-05-12

**Authors:** Disha P. Vala, Bhavesh N. Socha, Victoria G. Collins, Mehul P. Parmar, Chirag D. Patel, Savan S. Bhalodiya, Subham G. Patel, Sourav Banerjee, Hitendra M. Patel

**Affiliations:** a Department of Chemistry, Sardar Patel University Vallabh Vidyanagar-388 120 Gujarat India hm_patel@spuvvn.edu; b Department of Materials sciences, Sardar Patel University Vallabh Vidyanagar-388 120 Gujarat India; c Division of Cancer Research, School of Medicine, University of Dundee Dundee DD1 9SY UK s.y.banerjee@dundee.ac.uk

## Abstract

In this study, we successfully synthesized a series of pro-chiral 2-acetyl-*N*-aryl-2-(prop-2-yn-1-yl)pent-4-ynamide/-2-allyl-4-enamide derivatives 5(a–n) starting from 4-enamide-*N*-(prop-2-yn-1-yl)pent-4-ynamide/-*N*-allyl-4-enamide 4 through a bimolecular nucleophilic substitution (S_N_2) reaction. We report, for the first time, a novel C–C bond formation between allyl and acetoacetanilide derivatives, supported by detailed mechanistic analysis involving alkyne interactions. The reaction mechanism, characterized by a nucleophilic attack at the substrate's carbon center leading to displacement of the leaving group, was validated through intrinsic reaction coordinate (IRC) analysis, providing deeper insights into its pathway and dynamics.

## Abstract 2

We synthesized a series of pro-chiral derivatives 5(a–n)*via* S_N_2 reaction, demonstrating a C–C bond formation between allyl and acetoacetanilides. Mechanistic validation through IRC analysis offers deeper insights into its pathway and dynamics.

## Graphical abstract

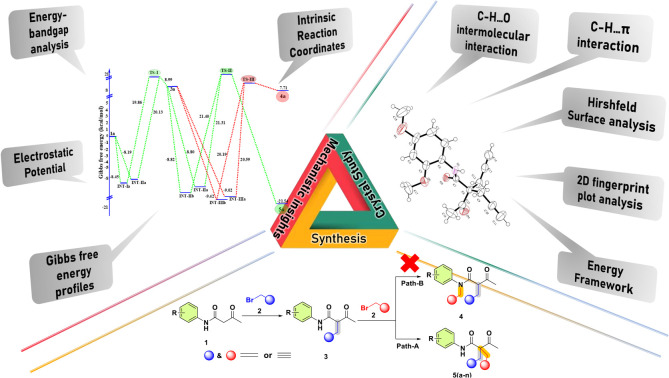

## 1 Introduction

Pro-chiral dienes/diynes are extensively implemented in various areas such as drug synthesis, agrochemicals, and pharmacy as well as in asymmetric organic synthesis as they also act as potential ligands in many metal-complex formations.^1^ A library of pro-chiral diynes/dienes with high structural diversity is useful for reaction development.^2^ The acetylene group has numerous elaboration options, such as nucleophilic additions, cyclizations, and metal-catalyzed coupling reactions ensuing optically active pro-chiral synthons are extremely important.^3,4^ Innovative approaches, such as diastereoselective desymmetrization, have enabled the transformation of symmetric dienes into chiral building blocks with high stereochemical control.^5,6^ The development of chiral pincer complexes has transformed enantioselective processes such as hydrogenation and alkynylation, while the stereoselective production of conjugated dienes has opened up new avenues for their application in polymers and materials science.^7^ Furthermore, photoredox catalysis and biocatalysis are gaining popularity because of their environmentally friendly and precise methods for producing chiral compounds.^8–10^ Computational chemistry has made a significant contribution by refining synthetic techniques, predicting reaction outcomes, and studying molecular orbitals, thereby driving reaction development.^11^ These breakthroughs pave the way for the development of a structurally diverse library of pro-chiral dienes and diynes, which will spur innovation in medicine, agrochemical, and material research.^12^ Although acetylene groups can be chemically modified to create optically active pro-chiral synthons, enantioselective catalytic synthesis is challenging owing to a lack of efficient ways for forming stereocenters with an acetylene group.^13–15^ The S_N_2 reaction is important and useful in organic transformation.^16^ Computational chemistry has emerged as an extremely valuable tool in organic chemistry^17^ for learning and identifying reaction intermediates and transition states, evaluating bond distances and atomic charges,^18^ and analyzing molecular orbitals. Furthermore, energy calculations aid in understanding the thermodynamic and kinetic factors of a reaction coordinate.^19^

In continuation of our previous work,^20–25^ herein, we synthesised pro-chiral diynes/dienes 5(a–n) using acetoacetanilide derivatives 1(a–g) and propargyl/allyl bromide 2(a–b). We synthesised 14 analogues of pro-chiral allyl and alkyne containing acetoacetanilide 5(a–n) through the –NH functionalization of acetoacetanilides. Density functional theory (DFT) studies were conducted to understand the reaction mechanism, highlighting that the –NH moiety is less active in the reaction pathway than the –CH moiety.

## 2 Results and discussion

### 2.1 Chemistry

**Scheme 1:**
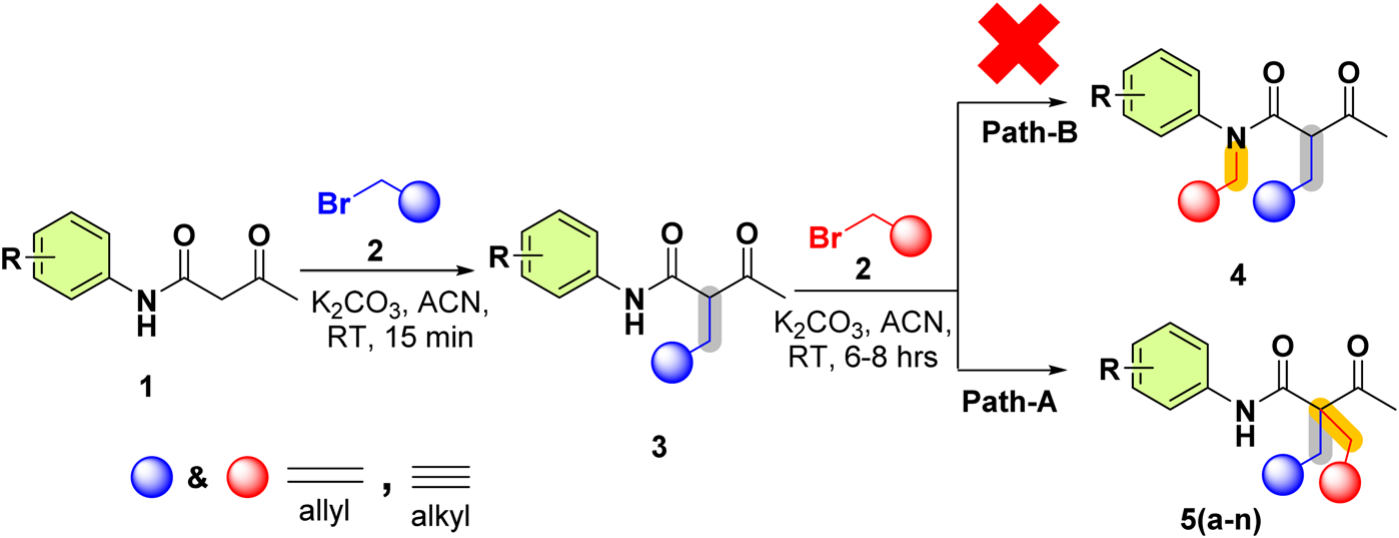
Synthesis of -acetyl-*N*-aryl-2-(prop-2-yn-1-yl)pent-4-ynamide/-2-allyl-4-enamide 5(a–n) over 4-enamide-*N*-(prop-2-yn-1-yl)pent-4-ynamide/-*N*-allyl-4-enamide.

These pro-chiral diynes/dienes were synthesised using acetoacetanilide derivatives 1(a–g) and propargyl/allyl bromide 2(a–b). In this transformation (Scheme 1), the base first deprotonates the active methylene –H of acetoacetanilide 1(a–g), leading to the *in situ* formation of a racemic mixture of 2-acetyl-*N*-phenylpent-4-enamide/-ynamide 3, which immediately undergoes for the reaction led to the pro-chiral diynes/dienes 5(a–n) instead of deprotonation from the amide proton (–NH–C=O) 4, as well as the mechanistic approach of both pathways (A & B). To confirm its formation and structure, 3 was also isolated and characterized using NMR and Mass spectroscopy. This S_N_2 transformation was carried out for better understanding reaction pathways, stability of intermediates and transition states. DFT studies were performed to elucidate the reaction mechanism, revealing why the –NH moiety does not participate as actively in the reaction pathway compared to the –CH moiety.

For optimization, acetoacetanilide 1a and propargyl bromide 2a were initially treated in the presence of K_2_CO_3_ (Table 1), which gave multiple spot-on TLC analyses (Entries 1 & 2). CS_2_CO_3_ also led to a mixture (Entry 3). Subsequently, 2 equiv. of 2a were taken. After 30 min, a mixture of products was generated and acetoacetanilide was still unreacted (Entry 4). After 10 hours, the reaction gave a single spot (Entry 5). 3 equiv. of 2a led to the single spot after 10 hours (Entry 6) (Table 1).

**Table 1:** Optimization of reaction conditions^a^^b^^c^

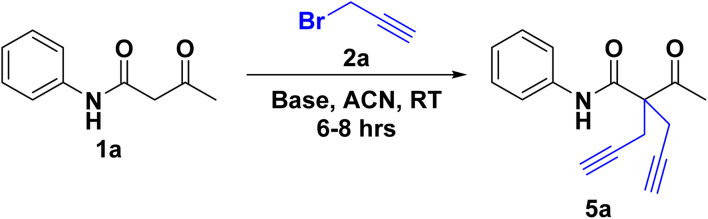					

Entry	Base	Amount of the base	Amount of 2a	Time (min) t	% Conversion of 1a
1	K_2_CO_3_	2 equiv.	1 equiv.	30 min	Mixture
2	K_2_CO_3_	2 equiv.	1 equiv.	18 h	Mixture
3	Cs_2_CO_3_	1.5 equiv.	1 equiv.	18 h	Mixture
4	K_2_CO_3_	2 equiv.	2 equiv.	30 min	Mixture
5	K_2_CO_3_	2 equiv.	2 equiv.	10 h	100%
6	K_2_CO_3_	3 equiv.	3 equiv.	10 h	100%

a Reaction condition: 1a (1 mmol), 2a, base, 5–7 ml ACN, RT.

b Observed from TLC analysis.

c Isolated yield.

**Scheme 2:**
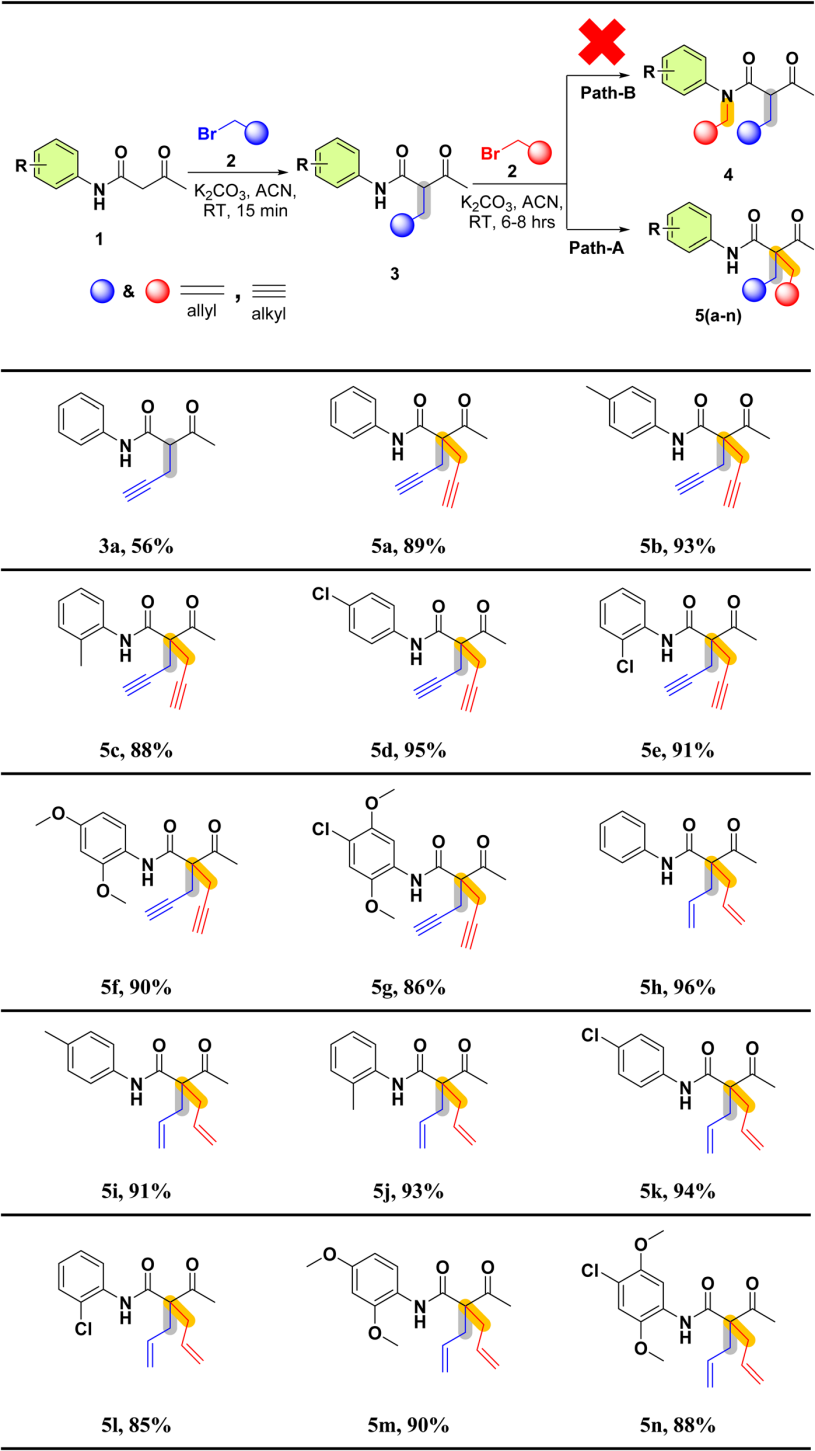
Substrate scope of 2-acetyl-*N*-aryl-2-(prop-2-yn-1-yl)pent-4-ynamide/-2-allyl-4-enamide 5(a–n).

The reaction of 2(a–b) with different acetoacetanilide 1(a–g) using commercially available K_2_CO_3_ achieved 100% conversion yield for 1a within 8–10 h using acetonitrile. The desired crude product precipitated out with water after stirring for 1–1.5 h. This crude product was filtered and washed first with (2 × 20 mL) water, followed by (2 × 10 mL) *n*-hexane. All acetoacetanilide derivatives gave excellent yields (Scheme 2). First, the facial synthesis of the reaction between acetoacetanilide 1a and propargyl bromide 2a in the presence of K_2_CO_3_ for the generation of compound 3a, which further reacted with another molecule of propargyl bromide, led to the formation of prochiral dialkyne 5a.

**Scheme 3:**
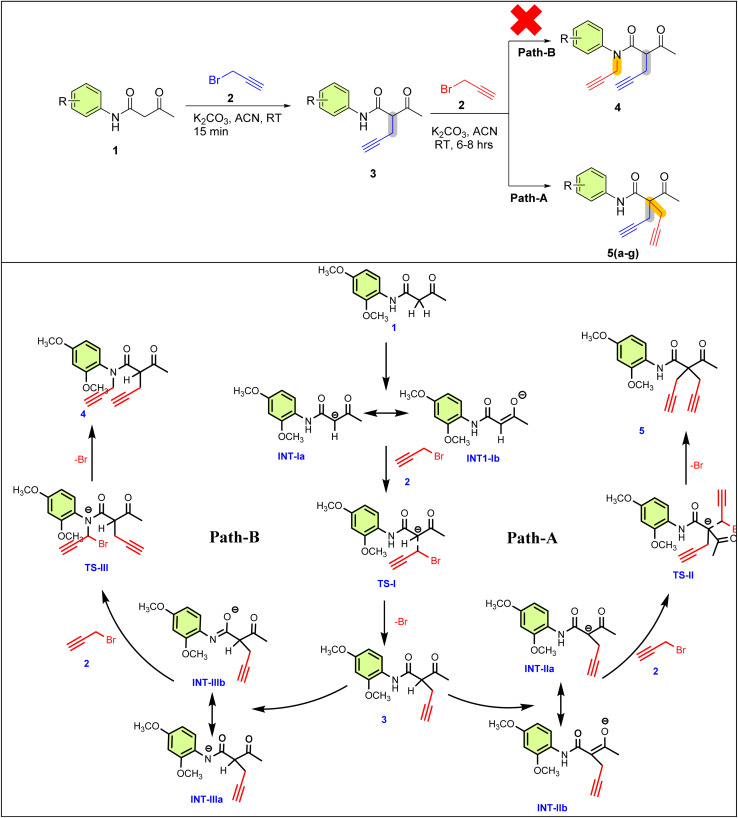
Mechanism for possible pathways for active and inactive sites.

Here, K_2_CO_3_ deprotonates the nucleophile, increasing the nucleophilicity as it converts into the more reactive conjugate-base, which are the keto–enol tautomers INT-Ia & INT-Ib (Scheme 3). This Nu-attacks the electrophilic carbon of 2, where the carbon is partially bonded to both the nucleophile and the leaving group (-Br)-formed transition state-I. -Br then departs, resulting in the formation of 3. Subsequently, these 3 further undergo S_N_2 reaction, resulting in 5 and 4. Here, we explore the reaction mechanism of the formation of 5 and 4*via* DFT using Gaussian 09.^26^

### 2.2 Crystallographic investigation

**Fig. 1:**
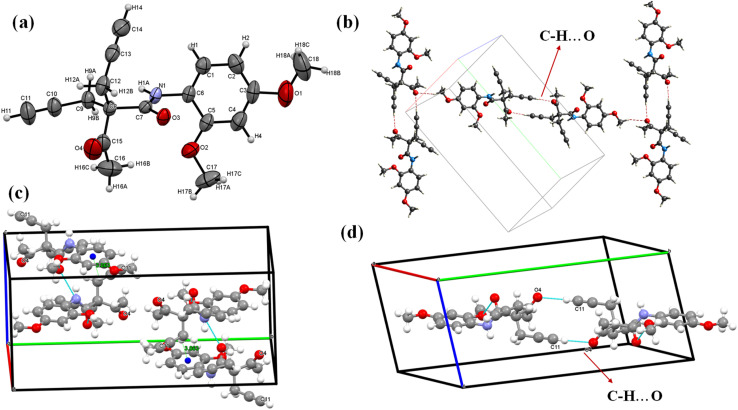
(a) ORTAP view of the 5f molecule with 50% probability, (CCDC 2337812). (b) C–H⋯O intermolecular interaction, (c) C–H⋯π interaction and the (d) graph set motif.

For the structure–function relationship, single crystals of the final product 5f were obtained using a slow evaporation method (Fig. 1a). The crystal crystallized in the monoclinic system with space group *P*2_1_/*c*, comprising four asymmetric molecules per unit cell (Table S1†). The two allyl moieties were arranged in such a way that one moiety was displaced by an angle of 110° and then rotated around an angle of 109°, with torsional angles of approximately −25.69° and 7.27°. The intra and intermolecular interactions, involving N–H⋯O, O–H⋯O, and C–H⋯O hydrogen bonds, played a critical role in stabilizing the molecule (Fig. 1b). The oxygen atoms facilitated binding between four molecules in the unit cell *via* intermolecular interactions, including C9–H9A⋯O9, N1–H1A⋯O3, and C18–H18⋯O4. In addition, the two C–H⋯O interactions between two 5f molecules form the graph set motif (Fig. 1d). The crystal structure was stabilized *via* carbon atoms (C9 and C17) from the allyl moieties and another carbon from the O–CH_3_ group on the ring, which participate in a C–H⋯π interaction (Fig. 1c), where the π system is sandwiched between two other molecules positioned above and below the π ring (Fig. S27†).

### 2.3 Computational analysis

#### 2.3.1 Optimization

Density functional theory (DFT) calculations were carried out to complement the experimental data and provide in-depth insights into the reaction mechanism of the reactions, electronic structure, and thermodynamic properties of the molecules. The Gibbs free energy change (Δ*G*) of the reaction was computed to evaluate the thermodynamic feasibility and spontaneity of the transformation. To further explore the reaction pathway, we used the intrinsic reaction coordinate (IRC) method to confirm the transition states and understand the energetic profile along the reaction coordinate. Electrostatic potential (ESP) surfaces were analyzed to visualize the charge distribution and predict the reactive sites. The HOMO–LUMO energy gaps were also calculated to examine the electronic and chemical nature of the intermediates states and products. Finally, thermal and entropy corrections were incorporated to assess the overall thermodynamic profile of the reaction.

All geometry optimizations and frequency calculations were performed using the B3LYP functional with the 6–311++G(d,p) basis set. The ‘++’ indicates that diffuse functions were included on all atoms, which is particularly important for accurately modeling the anionic species and long–range interactions. Solvent effects were incorporated using the PCM solvation model with acetonitrile as the solvent [SCRF = (solvent = acetonitrile)]. The optimization of the molecular structures using DFT showed excellent convergence for most of the systems, with all key parameters well within the defined thresholds. The mean maximum force across the systems was approximately 0.00003, significantly below the threshold of 0.000450. The RMS force also averaged at around 0.00052, comfortably meeting the threshold of 0.000300. For displacements, the mean maximum displacement was calculated at 0.00102, which was well within the allowable limit of 0.001800. The mean RMS displacement was 0.00067, which also satisfied the threshold of 0.001200. These results indicate successful structural optimization for the majority of the systems, with the exception of a few intermediates (INT-IIa, INT-IIb, INT-IIIb) and a transition state (TS-III), where slight deviations in force and displacement values were noted. Despite these exceptions, the overall convergence performance was robust and in line with the expectations for accurate molecular structure optimization. Solvent effects were incorporated using the Polarizable Continuum Model (PCM) with acetonitrile as the solvent *via* the scrf = (solvent = acetonitrile) approach (Table S3†).

#### 2.3.2 Electrostatic potential

The electrostatic potential (ESP) of the molecules is presented in the ESI File (Fig. S28†). The ESP surface shows that oxygen and nitrogen atoms are more electronegative, while carbon and hydrogen are more electropositive. Notably, when the parent linker transitions to the intermediate (INT) and transition (TS) states, the highly positive potential shifts to a slightly negative potential. This change occurs due to the redistribution of the electron density during bond formation and breakage, creating localized negative potentials that indicate increased reactivity. These regions can attract positively charged species, underscoring their importance in the reaction mechanism.^27,28^

#### 2.3.3 Energy-bandgap analysis

**Fig. 2:**
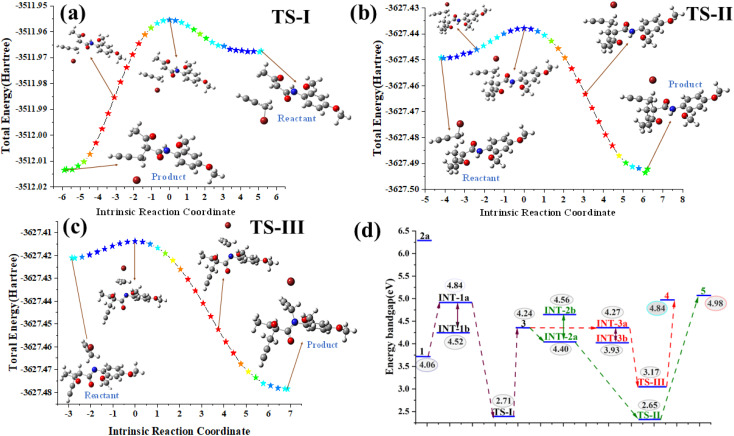
(a–c) Intrinsic reaction coordinate (IRC) pathways illustrating the progression from the reactant to product through various transition states (TS-I-TS-III); (d) band-gap analysis.

The HOMO–LUMO energy gaps and related quantum chemical parameters are provided in the ESI.† The differences in energy gaps between the reactant and product reflect the changes in the electronic structure during the transition from the intermediates to the final products (Fig. 2d). Compound 4 exhibits a HOMO–LUMO gap of 4.84 eV, while 5 shows a slightly higher gap of 4.98 eV, indicating a potential decrease in the chemical reactivity. Transitions from intermediates INT-IIa and INT-IIb highlight the key pathways to the most reactive configurations. Transition states TS-I (c and TS-II (2.65 eV) exhibit lower HOMO–LUMO gaps compared to TS-III (3.18 eV), suggesting that TS-II may be more electronically favorable for progression toward the final products. A smaller HOMO–LUMO gap can indicate increased reactivity, which may facilitate the reaction pathway, making TS-II the most favorable transition states based on this analysis.^29,30^ In terms of electronegativity (*χ*), molecule 5 exhibits a value of 3.61 eV, which is lower than that of 4 at 3.74 eV. This reduced electronegativity may contribute to the enhanced robustness of 5. Furthermore, the chemical hardness (*η*) for 5 is measured at 2.49 eV, slightly higher than that for 4 (2.42 eV). This suggests that 5 is marginally harder, indicating a greater resilience to external perturbations. Moreover, the electrophilicity (*ω*) values reinforce this trend; 5 has a lower electrophilicity of 5.14 eV compared to 4 5.52 eV. This lower value supports the argument for 5 increased stability. Additionally, the maximum charge transfer (Δ*N*_max_) is noteworthy: while 4 has a Δ*N*_max_ of 1.54, 5 shows a value of 1.45. This indicates a lesser tendency for charge transfer in 5, further confirming its superior stability during chemical processes (Table S2†).^31–33^

#### 2.3.4 Intrinsic reaction coordinates

In this study, we explore the intrinsic reaction coordinate (IRC) for three key TS (TS-I, TS-II, and TS-III) associated with the reaction pathway. The reactant-to-product pathways are shown in Fig. 2a–c. For TS-I, the activation energy is −1.63 kcal mol^−1^, indicating that this step can proceed without a significant energy barrier, facilitating a smooth transition during the reaction. Similarly, for TS-II, we observe an identical activation energy of −1.63 kcal mol^−1^, reinforcing its accessibility and favorability as a subsequent step in the reaction pathway. In contrast, for TS-III, the activation energy is −1.00 kcal mol^−1^, suggesting that this transition requires slightly more energy compared to TS-I and TS-II. Among the TS, TS-I and TS-II exhibit identical activation energies, making them equally favorable for the reaction, and both follow an exothermic pathway. This exothermic nature is crucial as it indicates that the reaction releases energy, contributing to the overall thermodynamic stability of the products. Importantly, the reaction is expected to follow the pathway from TS-I to TS-II, rather than TS-I to TS-III. This preference is based on the comparable and lower activation energies of TS-I and TS-II, which makes this route more thermodynamically favorable. In contrast, the pathway to TS-III, with a slightly greater activation energy, suggests a greater energy requirement, which could hinder its accessibility and make it a less favorable route. The IRC pathways, illustrated in Fig. 2a–c, clearly depict the energy landscape associated with each transition state.^34^

#### 2.3.5 Gibbs free energy profile

**Fig. 3:**
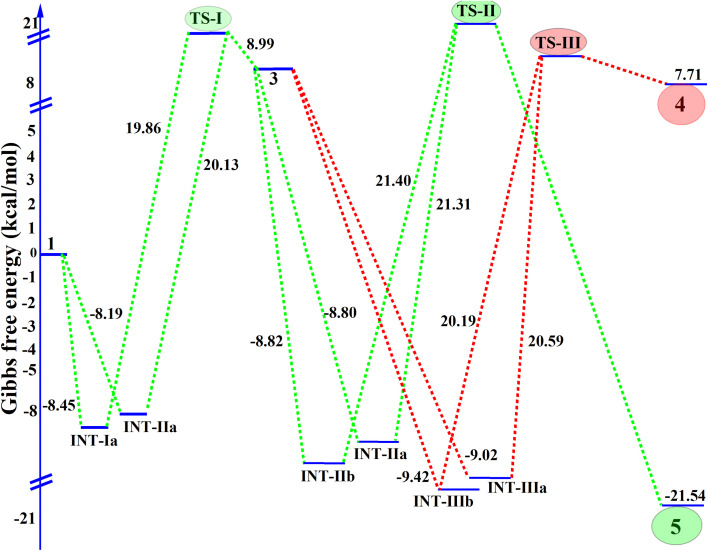
Gibbs free energy profile (kcal mol^−1^) of the active and inactive reaction molecules, along with the activation energy for the conversion of reactants into the final product.

The Gibbs free energy profile corresponding to the proposed reaction mechanism is presented in Fig. 3, providing a detailed understanding of the reaction pathway and the stability of the intermediates, in correlation with the single-crystal data. The reaction starts with acetoacetanilide (1), set as the reference point with a Gibbs free energy of 0.00 kcal mol^−1^, which undergoes alkylation with two moles of propargyl bromide (2) in the presence of K_2_CO_3_. This initial step leads to the formation of two possible intermediates: INT-1a and INT-1b, with the corresponding Gibbs free energies of −8.45 kcal mol^−1^ and −8.19 kcal mol^−1^, respectively. Among these, INT-1a is more thermodynamically stable due to its lower free energy, indicating that the reaction preferentially proceeds through this pathway.^35,36^ The transition from INT-1a to the first transition state TS-I requires overcoming an energy barrier of 19.86 kcal mol^−1^, slightly lower than the 20.13 kcal mol^−1^ needed for the transition from INT-1b to TS-I. This slight difference further confirms that the INT-1a → TS-I route is energetically more favorable, reinforcing the dominance of the INT-1a pathway in the reaction mechanism. Following this, the key intermediate 3 undergoes a second transformation involving an additional mole of propargyl bromide (2) and K_2_CO_3_, leading to four potential intermediates: INT-IIa (−8.80 kcal mol^−1^), INT-IIb (−8.82 kcal mol^−1^), INT-IIIa (−9.02 kcal mol^−1^), and INT-IIIb (−9.42 kcal mol^−1^). Although INT-IIIb exhibits the lowest energy among these species, it does not lie along the main reaction coordinate. The reaction proceeds through INT-IIa, which transitions *via* the second transition state TS-II, requiring an activation barrier of 21.40 kcal mol^−1^. This pathway leads to the formation of the final product 5, with a significantly stabilized Gibbs free energy of −21.54 kcal mol^−1^. An alternate route through INT-IIIa and TS-III ultimately leads to product 4, which is thermodynamically less favorable with a final energy of 7.71 kcal mol^−1^. Despite similar activation energies, the substantial energy difference between the final products clearly establishes that the TS-II → 5 pathway is more favorable, both kinetically and thermodynamically (Table S4†).

Overall, the computational energy profile strongly supports the reaction pathway 1 → INT-1a → TS-I → 3 → INT-Iia → TS-II → 5 as the dominant and most stable route. This mechanistic insight is further corroborated by experimental observations, including single-crystal X-ray data and electronic structure analyses, thereby providing a comprehensive understanding of the reaction's progression and the stability of the key species involved.^37,38^

## 3 Intra and intermolecular interaction studies

### 3.1 Hirshfeld surface analysis

**Fig. 4:**
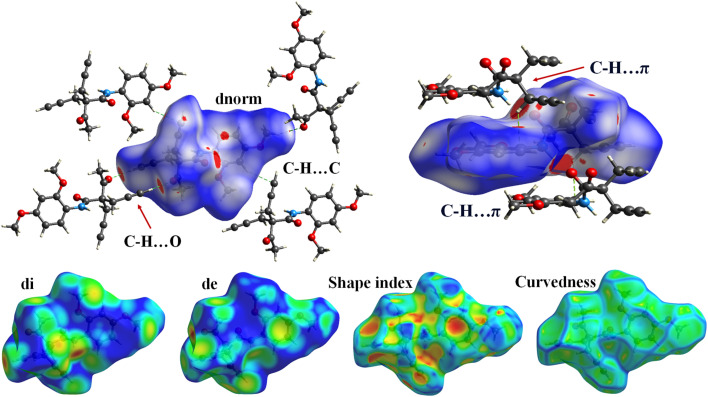
Hirshfeld surface analysis of the 5f molecule: dnorm, di, de, shape index and curvedness.

The Hirshfeld surface analysis was carried using the Crystal Explorer 17.5 software with the same method and basis set that was used in DFT calculation. The Hirshfeld surface analysis of molecule 5f provides a comprehensive understanding of the intermolecular interactions governing its crystal packing (Fig. 4). The dnorm surface reveals significant red spots near the oxygen atoms and some carbon atoms, indicating regions of close intermolecular contacts, such as hydrogen bonding or van der Waals interactions. Additionally, a small red area is observed near the nitrogen atom, signifying its involvement in weaker interactions. The rest of the molecule displays white and blue regions, corresponding to neutral and less significant contacts, respectively. On the di surface, the nitrogen and carbon atoms exhibit prominent red spots, further corroborating their roles as interaction sites. Conversely, the de surface highlights red areas primarily around the oxygen atoms, emphasizing their accessibility and participation in intermolecular interaction. The shape index surface analysis identifies red and blue triangular patterns on the carbon atoms that are not part of the benzene ring, indicative of these regions' predisposition to participate in C–H⋯π interactions. This observation suggests that these carbons play a critical role in stabilizing the molecular assembly through π-cloud interactions. The curvedness surface analysis shows flat areas corresponding to the benzene ring in molecule 5f. These flat regions suggest a high propensity for π⋯π stacking interactions, which are essential for the molecule's crystal stability and packing. The Hirshfeld surface analysis thus elucidates the intricate balance of intermolecular forces in molecule 5f, providing valuable insights into its structural behavior. The analysis revealed a globularity value of 0.286 and an asphericity of 0.324, indicating that the voids possess a slightly irregular and non-spherical geometry. These results highlight the anisotropic nature of the pores, which may contribute to selective guest inclusion and functional versatility.

**Fig. 5:**
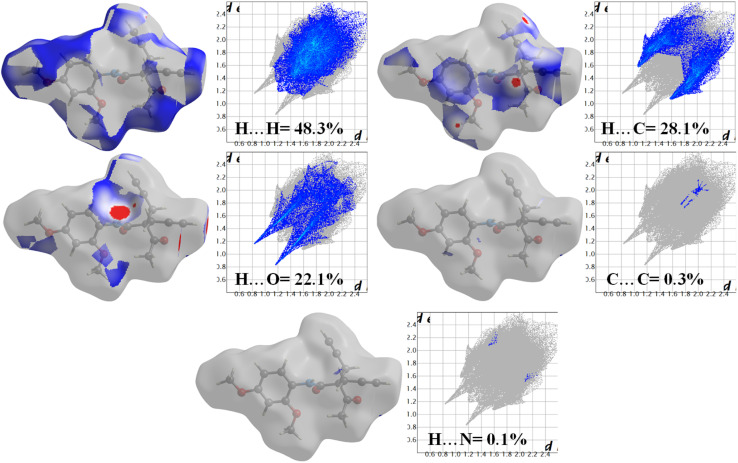
2D Fingerprint plot with the qualitative interaction % contribution in molecular packing.

In the 2D fingerprint plot analysis, the interaction contributions of various atomic pairs are represented in the form of distinctive wings. The plot reveals that hydrogen–hydrogen (H⋯H) interactions dominate the molecular interactions, contributing 48.3% to the total interactions. Hydrogen-carbon (H⋯C) interactions are also significant, accounting for 28.1%. The hydrogen-oxygen (H⋯O) interactions show a contribution of 22.1%, which is prominently displayed as a distinct wing in the plot. These H⋯O interactions are particularly important as they suggest potential hydrogen bonding or other intermolecular interactions that could influence the overall stability and properties of the system. Carbon–carbon (C⋯C) and hydrogen-nitrogen (H⋯N) interactions have minimal contributions, with 0.3% and 0.1%, respectively (Fig. 5).

### 3.2 Energy framework

**Fig. 6:**
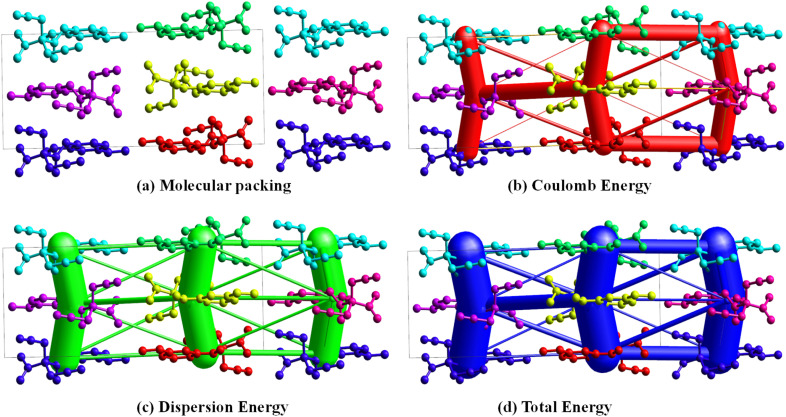
Three energy frameworks of 5a molecular with molecular packing with visualization of interaction strength (a) molecular packing, (b) Coulomb energy, (c) dispersion energy and (d) total energy.

The intermolecular interaction energies were calculated using the B3LYP/6-311G(d,p) energy model available in Crystal Explorer 17.5, where a cluster of molecules is generated by applying crystallographic symmetry operations with respect to a selected central molecule within the radius of 3.8 Å by default. The total intermolecular energy *E*_tot_ is the sum of electrostatic *E*_ele_, polarization *E*_pol_, dispersion *E*_dis_ and exchange-repulsion *E*_rep_ energies, respectively. The molecular packing, Coulomb energy, dispersion energy and total energy are shown in Fig. 6a–d, respectively. The thickness of the tubes correlates with the magnitude of the interaction energy. The tube thickness is large toward the −*x*, −*y*, −*z* symmetry molecular axes. Two symmetry-related molecules participate in interactions with the original molecule *via* C–H⋯O hydrogen bonds one involving the propyne moiety and the other from the acetyl group (Fig. 6d).

**Fig. 7:**
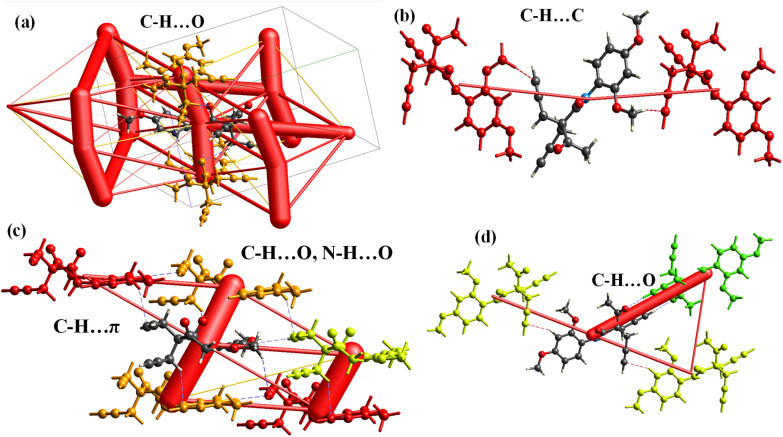
Coulombic energy analysis of various intermolecular interactions and their respective interaction strengths. (a) C–H⋯O interaction, where the C–H group (donor) from a symmetry-related molecule interacts with an oxygen atom from the original molecule (*E*_dis_ = –17.1 kcal mol^−1^), along with an N–H⋯O (amide) interaction. (b and c) C–H⋯π interactions between the aryl ring of a symmetry-related molecule and a C–H group from the original molecule (*E*_dis_ = –4.13 kcal mol^−1^). (d) Intermolecular C–H⋯O interaction between a C–H group from the acetyl moiety and the oxygen atom of a carbonyl group.

Energy framework analysis reveals key intermolecular interactions contributing to the stability of the crystal structure. Table 2 highlights the interactions, symmetry operations, and corresponding energy components, including electrostatic (*E*_ele_), polarization (*E*_pol_), dispersion (*E*_dis_), repulsion (*E*_rep_), and total interaction energy (*E*_tot_). For the interaction at *R* = 9.57 Å, symmetry operation *x*, −*y* + 1/2, *z* + 1/2 describes a C–H⋯C interaction involving an aryl ring. This interaction exhibits a moderate dispersion energy (*E*_dis_ = −4.13 kcal mol^−1^) and repulsion (*E*_rep_ = 2.0 kcal mol^−1^), resulting in a total energy of *E*_tot_ = −3.75 kcal mol^−1^ (Fig. 7b). The second interaction, also at symmetry operation *x*, −*y* + 1/2, *z* + 1/2 with *R* = 4.53 Å, involves a combination of C–H⋯O, N–H⋯O (amide), and C–H⋯π (prop-2-yne moiety) interactions. This interaction is characterized by strong electrostatic (*E*_ele_ = −2.46 kcal mol^−1^) and dispersion (*E*_dis_ = −17.11 kcal mol^−1^) contributions, resulting in the most significant total interaction energy (*E*_tot_) of −18.71 kcal mol^−1^. The strong electrostatic nature of this interaction, supported by substantial dispersion contributions, underscores its importance in stabilizing the crystal (Fig. 7c).

**Table 2:** Intermolecular interactions, symmetry operations, distances (*R*), and energy components (*E*_ele_, *E*_pol_, *E*_dis_, *E*_rep_ and *E*_total_) of the crystal structure. The strongest interaction is at *R* = 4.53 Å with *E*_tot_ = −18.71 kcal mol^−1^

*N*	Symop	*R*	*E*_ele	*E*_pol	*E*_dis	*E*_rep	*E*_tot	Interactions
2	*x*, −*y* + 1/2, *z* + 1/2	9.57	−1.07	−0.33	−4.13	2.0	−3.75	C–H⋯C (aryl ring)
2	*x*, −*y* + 1/2, *z* + 1/2	4.53	−10.32	−3.60	−17.1	15.82	−18.71	C–H⋯O, N–H⋯O (an amide) and C–H⋯π (prop-2-yne moiety)
1	−*x*, −*y*, −*z*	12.39	−6.81	−1.33	−3.03	6.78	−6.64	C–H⋯O (prop-2-yne moiety) C–H⋯O (acetyl moiety)

Lastly, the interaction at *R* = 12.39 Å with symmetry operation −*x*, −*y*, −*z* features C–H⋯O interactions involving both the prop-2-yne and acetyl moieties. This interaction demonstrates a balance between electrostatic (*E*_ele_ = −6.8 kcal mol^−1^), dispersion (*E*_dis_ = −3.03 kcal mol^−1^), and repulsion energies (*E*_rep_ = 6.78 kcal mol^−1^), leading to a total energy of *E*_tot_ = −6.64 kcal mol^−1^. Among the analyzed interactions, the C–H⋯O, N–H⋯O (amide), and C–H⋯π (prop-2-yne moiety) interaction at *R* = 4.53 Å is the strongest, as evidenced by its highly negative total energy (*E*_tot_ = −18.71 kcal mol^−1^). This highlights the dominant role of hydrogen bonding and π interactions in driving the stability of the crystal lattice (Fig. 7b).

## 4 Conclusion

We synthesized a series of pro-chiral 2-acetyl-*N*-subtituted-phenyl-2-(prop-2-yn-1-yl)pent-4-ynamide/-2-allyl-4-enamide analogues 5(a–n) using a K_2_CO_3_ promoted S_N_2 reaction in ACN at RT. The reaction of diverse acetoacetanilides 1(a–g) with propargyl/allyl bromide 2(a–b) efficiently achieved an excellent yield of up to 96%, a significant contribution to the field of asymmetric synthesis with fascinating implications for future applications. This work not only gives insight into the reaction pathways, but also lays the groundwork for the construction of structurally diverse libraries of pro-chiral compounds, using approaches such as S_N_2 reactions under optimal circumstances and DFT calculations. DFT calculations provided further insight into the reaction path of formation of 5(a–n), which undergoes S_N_2 to give a pro-chiral diyne/diene product instead of deprotonation from the amide proton (–NH–C=O). The reaction mechanism, structural stability, and energy profile of INT & TS were thoroughly examined using the DFT platform, single crystal analysis, and energy band gap evaluation compilations with synthetic compounds. Slow evaporation yielded well-defined single crystals of product 5f, crystallized in the monoclinic system, stabilized by intermolecular interactions including hydrogen bonds and C–H⋯π interactions. DFT optimizations confirmed structural stability across intermediates, with notable favorability observed in the INT-1a pathway due to the Gibbs free energy differences. The Gibbs free energy profile illustrates the stability of the intermediate and unstable molecules during the transition to the final product, along with the activation energy for the conversion from reactants to the final product. Transition states TS-I and TS-II exhibited identical low activation energies, confirming an energetically favorable pathway toward 5 as the most stable product. The electrostatic potential analysis highlighted critical reactive sites, while band gap differences reinforced 5's thermodynamic stability over 4, corroborating crystallographic and theoretical findings. Consequently, desymmetrization of these pro-chiral diynes/dienes to 1,2,3-triazole will be carried out.

## Data availability

The data supporting this article, including DFT analysis carried out using Gaussian 09,^26^ are included as part of the ESI.† Crystallographic data for compound 5f have been deposited at the CCDC under 2337812.

## Conflicts of interest

The authors declare no competing interests.

## Supplementary Material

RA-015-D5RA02166A-s001

RA-015-D5RA02166A-s002
