# Supplementary material for: Insights into the synthesis and structural properties of pro-chiral 2-acetyl-N-aryl-2-(prop-2-yn-1-yl)pent-4-ynamides/-2-allyl-4-enamide derivatives through kinetics and energy frameworks
Source: RSC Adv. 2025 May 12;15(20):15712–23. doi: 10.1039/d5ra02166a (PMC12068048; doi:10.1039/d5ra02166a)
Supplement: RA-015-D5RA02166A-s001 [file RA-015-D5RA02166A-s001.pdf]

Supplementary Information (SI) for RSC Advances.
This journal is © The Royal Society of Chemistry 2025

Supporting Information for

Synthesis and Structural Insights of Pro-Chiral 2-acetyl-*N*-aryl-2-(prop-2-yn-1-yl)pent-4-ynamides/-2-allyl-4-enamide derivatives through Kinetics and Energy Frameworks

Disha P. Vala[a], Bhavesh N. Socha[b], Victoria G. Collins[c], Mehul P. Parmar[a], Chirag D. Patel[a], Savan S. Bhalodiya[a], Subham G. Patel[a], Sourav Banerjee[c]*, and Hitendra M. Patel[a]*

[a] Department of Chemistry, Sardar Patel University, Vallabh Vidyanagar-388 120, Gujarat, India.

[b] Department of Materials sciences, Sardar Patel University, Vallabh Vidyanagar-388 120, Gujarat, India.

[c] Division of Cancer Research, School of Medicine, University of Dundee, Dundee DD1 9SY, UK

*Co-corresponding author: Sourav Banerjee, E-mail: s.y.banerjee@dundee.ac.uk

*Corresponding author: Hitendra M. Patel, E-mail: hm_patel@spuvvn.edu

Table of Contents

1 General Methods 2

1.1 Experimental techniques 2

1.2 Analytical 2

2 Materials and Methods 3

2.1 General Procedures for Synthesis of Pro-Chiral 2-acetyl-*N*-aryl-2-(prop-2-yn-1-yl)pent-4-ynamides/-2-allyl-4-enamides **5(a-n)** 3

3 Analytical data of the synthesized compounds 3

3.1 2-acetyl-*N*-phenylpent-4-ynamide **(3a)** 3

3.2 2-acetyl-*N*-phenyl-2-(prop-2-yn-1-yl)pent-4-ynamide **(5a)** 3

3.3 2-acetyl-2-(prop-2-yn-1-yl)-*N*-(*p*-tolyl)pent-4-ynamide **(5b)** 4

3.4 2-acetyl-2-(prop-2-yn-1-yl)-*N*-(*o*-tolyl)pent-4-ynamide **(5c)** 4

3.5 2-acetyl-*N*-(4-chlorophenyl)-2-(prop-2-yn-1-yl)pent-4-ynamide **(5d)** 4

3.6 2-acetyl-*N*-(2-chlorophenyl)-2-(prop-2-yn-1-yl)pent-4-ynamide **(5e)** 4

3.7 2-acetyl-*N*-(2,4-dimethoxyphenyl)-2-(prop-2-yn-1-yl)pent-4-ynamide **(5f)** 4

3.8 2-acetyl-*N*-(4-chloro-2,5-dimethoxyphenyl)-2-(prop-2-yn-1-yl)pent-4-ynamide **(5g)** 4

3.9 2-acetyl-2-allyl-*N*-phenylpent-4-enamide **(5h)** 4

3.10 2-acetyl-2-allyl-*N*-(*p*-tolyl)pent-4-enamide **(5i)** 5

3.11 2-acetyl-2-allyl-*N*-(*o*-tolyl)pent-4-enamide **(5j)** 5

3.12 2-acetyl-2-allyl-*N*-(4-chlorophenyl)pent-4-enamide **(5k)** 5

3.13 2-acetyl-2-allyl-*N*-(2-chlorophenyl)pent-4-enamide **(5l)** 5

4 NMR Spectra of 3a & 5(a-g) 5
5 Mass Analysis image 15
6 Crystallographic investigation 18
7 DFT Calculations 19
7.1 Optimization 19

1 General Methods

1.1 Experimental techniques

The nomenclature of the synthesized molecules is done according to IUPAC. Also, the numbering in the carbon chain is based on the position of the carbon atom.

All the reactions are performed in an open-air atmosphere. All the solid and liquid reagents and needles are added to the RBF under the open-air atmosphere.

Percentages (%) refers to mass percentage.

The calculated yields refer to the limiting reagent component.

Paraffin oil baths (Silicone oil baths) are used to record the melting points. The temperature is set and controlled by using an adjustable contact thermometer. Melting points (M.P.) of all solid compounds were determined by utilizing an open capillary tube method and were uncorrected.

Reagents

All the reagents were purchased from TCI, Sigma-Aldrich, and Sisco Research Pvt. Ltd. and used without further purification. The reaction was performed using the conventional heating method.

Solvents

Solvents from the given companies were used with the corresponding quality grades and used without further purification.

The following solvents were used in thin-layer chromatography (TLC) and smart flash chromatography: DMF, DMSO, Ethyl acetate, n-hexane, methanol, dichloromethane

1.2 Analytical

Thin Layer Chromatography (TLC)

The progress of all chemical reactions was monitored by Thin-layer chromatography. For this purpose, TLC, on aluminium plates pre-coated with F252 silica gel 60 by Merck was used as the stationary phase. TLC plates were analyzed under Visible light (λ =400 nm to 750 nm).

Nuclear Magnetic Resonance (NMR) Spectroscopy

NMR Spectra ^{1}H NMR & ^{13}C NMR were recorded on the Brucker Avance Neo 500 MHz/ 400 MHz & 128 MHz/ 101 MHz FT-NMR spectrometer with a proton noise decoupling mode with a standard 5 mm probe. The chemical shift values are given in δ (ppm) and the coupling constant (J) is provided in Hertz. For the solvent, deuterated DMSO-d6, the signal of solvents

was used in the ^{1}H NMR spectra (δ = 2.52 ppm) and ^{13}C NMR spectra (δ = 39 ppm) as an internal standard for calibration. The spectra were viewed by utilizing the Top Spin of the company Bruker. The following abbreviations were used for the clear assignment of the signals and the spin multiplicities: s = singlet, d = doublet, t = triplet, q = quartet, dd = double of doublet, td = triplet of doublet, m = multiplet.

Melting Points

Melting points of the synthesized solid products were recorded using an open capillary method and were uncorrected.

Mass Spectrometry

Mass spectra were recorded using MS (ESI-TOF) *m/z*.

Crystallization

For the sample preparation, 150 mg of the **(5f)** was dissolved in 100 ml of ethyl acetate : n-Hexane (7:3): 1drop DMSO in a beaker and heated until **5f** properly dissolved and the amount of solution was half (50 ml), after that 150 mg of activated charcoal was added to eliminate coloured impurities from the compound. Once the charcoal treatment solution was filtered, it was kept in a clean beaker covered with aluminium foil for a couple of days. When the solvent was evaporated, a single crystal of compound **5f** developed over 10-15 days.

2 Materials and Methods

2.1 General Procedures for Synthesis of Pro-Chiral 2-acetyl-*N*-aryl-2-(prop-2-yn-1-yl)pent-4-ynamides/-2-allyl-4-enamides **5(a-n)**

In RBF (50 ml) mixture of 1mmol *N*-(substituted phenyl)-3-oxobutanamide **1(a-g)**, 2.5 mmol Propargyl bromide **2(a-b)** and K_2CO_3 (4 equi.) were stirred at room temperature for 8-10 hrs in 5 ml ACN. At the end of the reaction (monitored by TLC technique), 20-30 ml water is added to the reaction mixture and stirred for 1-1.5 hrs. Precipitation of targeted product **5(a-n)** separated by simple filtration. The precipitate was washed thoroughly twice with water (2×10) and n-Hexane (2×10 ml) to afford the desired product in pure form.

3 Analytical data of the synthesized compounds

3.1 2-acetyl-*N*-phenylpent-4-ynamide **(3a)**

MS (ESI-TOF) *m/z* calcd For CHNO $(M + H)^+$: 215.10, Found: 215.18; % yield: 56%; MP: 124-126 °C; ^{1}H NMR (400 MHz, DMSO) δ 10.43 (s, 1H, -NH), 7.60 (d, *J* = 8 Hz, 2H, Ar-H), 7.33 (t, *J* = 8 Hz, 1H, Ar-H), 7.09 (t, *J* = 8 Hz, 1H, Ar-H), 3.85 (t, *J* = 8 Hz, 1H, -CH), 2.85 (t, *J* = 4 Hz, 1H, -C≡H), 2.63 - 2.60 (m, 2H, $-CH_2$), 2.20 (s, 3H, $-CH_3$); ^{13}C {1H}NMR (101 MHz, DMSO) δ 201.7 (-C=O), 165.8 (NH-<u>C</u>=O), 138.5, 128.7, 123.7, 119.3, 81.5, 72.4, 59.4, 28.3, 17.0.

3.2 2-acetyl-*N*-phenyl-2-(prop-2-yn-1-yl)pent-4-ynamide **(5a)**

MS (ESI-TOF) *m/z* calcd For CHNO $(M + H)^+$:253.11, Found: 253.19; % yield: 89%; ^{1}H NMR (500 MHz, DMSO-D6) δ 9.63 (s, 1H, -NH), 7.57 (d, *J* = 5 Hz, 2H, Ar-H), 7.32 (t, *J* = 5 Hz, 2H, Ar-H), 7.11 (t, *J* = 5 Hz, 1H, Ar-H), 3.04 (t, *J* = 5 Hz, 2H, -C≡H), 2.97 - 2.91 (m, 4H, -CH_2), 2.19 (s, 3H, $-CH_3$); ^{13}C {1H}NMR (126 MHz, DMSO-D6) δ 202.4 (-C=O), 166.6 (NH-<u>C</u>=O), 138.3, 128.6, 124.3, 120.9, 79.3, 74.5, 62.6, 25.9, 21.4.

3.3 2-acetyl-2-(prop-2-yn-1-yl)-*N*-(*p*-tolyl)pent-4-ynamide **(5b)**

MS (ESI-TOF) *m/z* calcd For CHNO $(M + H)^+$: 267.13, Found: 267.21; % yield: 93%; MP: 124-126 °C ^{1}H NMR (400 MHz, DMSO) δ 9.55 (s, 1H, -NH), 7.44 (d, *J*= 8 Hz, 2H, Ar-H), 7.12 (d, *J*= 8 Hz, 2H, Ar-H), 3.07 - 2.90 (m, 6H, Alkyne), , 2.25 (s, 3H, $-CH_3$), 2.18 (s, 3H, -CH_3); ^{13}C {1H}NMR (101 MHz, DMSO) δ 201.9 (-C=O), 166.0 (-NHC=O), 135.8, 133.3, 128.9, 121.0, 78.9 (C≡CH), 73.9 (C≡CH), 62.1 ($COCH_2CO$), 25.8 (CO-CH_3), 20.9 (Ph-CH_3), 20.0 (CH_2C≡CH);

3.4 2-acetyl-2-(prop-2-yn-1-yl)-*N*-(*o*-tolyl)pent-4-ynamide **(5c)**

MS (ESI-TOF) *m/z* calcd For CHNO $(M + H)^+$: 267.13, Found: 267.19; % yield: 88%; MP: 134-136 °C ^{1}H NMR (500 MHz, DMSO-D6) δ 9.43 (s, 1H, -NH), 7.24 - 7.09 (m, 4H, Ar-H), 3.02 (d, *J* = 20 Hz, 4H, $-CH_2$), 2.93 (t, *J* = 5 Hz, 2H, ≡CH), 2.24 (s, 3H, $-CH_3$), 2.17 (s, 3H, -CH_3); ^{13}C {1H}NMR (126 MHz, DMSO-D6) δ 202.6 (-C=O), 166.7 (NH-C=O), 135.9, 134.3, 130.3, 127.0, 126.6, 126.0, 79.43, 74.4, 62.1, 25.8 ($-CH_3$), 21.4 ($-CH_2$), 17.7 ($-CH_3$).

3.5 2-acetyl-*N*-(4-chlorophenyl)-2-(prop-2-yn-1-yl)pent-4-ynamide **(5d)**

MS (ESI-TOF) *m/z* calcd For CHNO $(M + H)^+$: 287.07, Found: 287.11; % yield: 95% MP: 110-112 °C ^{13}C {1H}NMR (126 MHz, DMSO-D6) δ 202.4 (-C=O), 166.6 (NH-C=O), 138.3, 128.6, 124.3 (Ar-C-Cl), 120.9, 79.3, 74.5, 62.6, 25.9, 21.4 ($-CH_3$).

3.6 2-acetyl-*N*-(2-chlorophenyl)-2-(prop-2-yn-1-yl)pent-4-ynamide **(5e)**

MS (ESI-TOF) *m/z* calcd For CHNO $(M + H)^+$: 287.07, Found: 278.11; % yield: 91%; MP: 108-110 °C

3.7 2-acetyl-*N*-(2,4-dimethoxyphenyl)-2-(prop-2-yn-1-yl)pent-4-ynamide **(5f)**

MS (ESI-TOF) *m/z* calcd For CHNO $(M + H)^+$: 313.13, Found: 313.34; % yield: 90%; MP: 108-110 °C ^{1}H NMR (500 MHz, DMSO-D6) δ 9.02 (s, 1H, -NH), 7.18 (d, *J* = 5 Hz, 1H, Ar-H), 6.61 (d, *J* = 5 Hz, 1H, Ar-H), 6.49 (d, *J* = 10 Hz, 1H, Ar-H), 3.75 (s, 3H, $-OCH_3$), 3.75 (s, 3H, $-OCH_3$), 2.96 (d, *J* = 10 Hz, 4H, $-CH_2$), 2.88 (t, *J* = 5 Hz, 2H, -C≡H), 2.22 (s, 3H, $-CH_3$); ^{13}C {1H}NMR (126 MHz, DMSO-D6) δ 202.7 (-C=O), 166.5 (NH-C=O), 158.3, 153.9, 126.7, 104.1, 98.8, 79.3, 74.2, 61.7, 55.5, 55.2, 25.6, 21.3.

3.8 2-acetyl-*N*-(4-chloro-2,5-dimethoxyphenyl)-2-(prop-2-yn-1-yl)pent-4-ynamide **(5g)**

MS (ESI-TOF) *m/z* calcd For CHNO $(M + H)^+$: 347.10, Found: 347.14; % yield: 86%; MP: 132-134 °C ^{1}H NMR (400 MHz, $CDCl_3$) δ 8.37 (s, 1H, -NH), 8.15 (s, 1H, Ar-H), 6.90 (s, 1H, Ar-H), 3.88 (s, 3H, $-OCH_3$), 3.85 (s, 3H, $-OCH_3$), 3.06 (d, *J* = 4 Hz, 4H, $-CH_2$), 2.37 (s, 3H, -CH_3), 2.10 (t, *J* = 4 Hz, 2H, -C≡H); ^{13}C {1H}NMR (101 MHz, $CDCl_3$) δ 204.6 (-C=O), 165.6 (NH-C=O), 149.1, 142.3, 126.3, 116.7, 112.5, 105.1, 78.6, 72.6, 63.5, 56.8, 56.6, 26.9, 22.7.

3.9 2-acetyl-2-allyl-*N*-phenylpent-4-enamide **(5h)**

MS (ESI-TOF) *m/z* calcd For CHNO $(M + H)^+$: 257.14, Found: 257.23; % yield: 96%; MP: 112-114 °C ^{1}H NMR (500 MHz, DMSO-D6) δ 9.50 (s, 1H, -NH), 7.57 (d, *J* = 5 Hz, 2H, Ar-H), 7.32, 7.30, 7.29 (t, *J* = 5 Hz, 2H, Ar-H), 7.09 (t, *J* = 5 Hz, 1H, Ar-H), 5.65 - 5.57 (m, 2H, -CH=C), 5.14 - 5.05 (m, 4H, $=CH_2$), 2.72 - 2.59 (m, 4H, $-CH_2$), 2.13 ($-CH_3$); ^{13}C {1H}NMR (126 MHz, DMSO-D6) δ 205.0 (-C=O), 169.1 (NH-C=O), 138.5, 133.0, 128.5, 124.0, 120.9, 118.9, 63.7, 35.4, 26.7 ($-CH_3$).

3.10 2-acetyl-2-allyl-*N*-(*p*-tolyl)pent-4-enamide **(5i)**

MS (ESI-TOF) *m/z* calcd For CHNO $(M + H)^+$: 271.16, Found: 271.11; % yield: 91%; MP: 106-108 °C ^{1}H NMR (500 MHz, DMSO-D6) δ 9.41 (s, 1H, -NH), 7.43 (d, *J* = 10 Hz, 2H, Ar-

H), 7.10 (d, *J* = 10 Hz, 2H, Ar-H), 5.64 - 5.56 (m, 2H, -CH=C), 5.14 - 5.05 (m, 4H, =CH_2), 2.71 - 2.58 (m, 4H, -CH_2), 2.25 (s, 3H, -CH_3), 2.12 (s, 3H, -CH_3); ^{13}C {1H}NMR (126 MHz, DMSO-D6) δ 205.0 (-<u>C</u>=O), 169.0 (NH-<u>C</u>=O), 136.0, 133.1, 133.0, 128.9, 121.0, 118.8, 63.6, 35.4, 26.7, 20.5.

3.11 2-acetyl-2-allyl-*N*-(*o*-tolyl)pent-4-enamide **(5j)**

MS (ESI-TOF) *m/z* calcd For CHNO (M + H)$^+$: 271.16, Found: 271.53; % yield: 93%; MP: 118-120 °C ^{1}H NMR (500 MHz, DMSO-D6) δ 9.27 (s, 1H, -NH), 7.24 - 7.12 (m, 4H, Ar-H), 5.70 - 5.62 (m, 2H, -CH=C), 5.19 - 5.10 (m, 4H, =CH_2), 2.73 - 2.61 (m, 4H, -CH_2), 2.18 (s, 3H, -CH_3), 2.17 (s, 3H, -CH_3); ^{13}C {1H}NMR (126 MHz, DMSO-D6) δ 205.19 (-<u>C</u>=O), 169.2 (NH-<u>C</u>=O), 136.0, 134.0, 133.1, 130.3, 126.9, 126.2, 126.0, 118.9, 63.0, 35.4, 26.7 (-CH_3), 17.9 (-CH_3).

3.12 2-acetyl-2-allyl-*N*-(4-chlorophenyl)pent-4-enamide **(5k)**

MS (ESI-TOF) *m/z* calcd For CHNO (M + H)$^+$: 291.10 Found: 291.17; % yield: 94%; ^{1}H NMR (500 MHz, DMSO-D6) δ 9.64 (s, 1H, -NH), 7.62 (d, *J* = 10 Hz, 2H, Ar-H), 7.36 (d, *J* = 5 Hz, 2H, Ar-H), 5.64 - 5.56 (m, 2H, -CH=C), 5.14 - 5.05 (m, 4H, =CH_2), 2.71 - 2.59 (m, 4H, -CH_2), 2.13 (s, 3H, -CH_3); ^{13}C {1H}NMR (126 MHz, DMSO-D6) δ 204.9 (-<u>C</u>=O), 169.3 (NH-<u>C</u>=O), 137.6, 133.0, 128.4, 127.6, 122.4, 118.9, 63.8, 35.4, 26.7 (-CH_3).

3.13 2-acetyl-2-allyl-*N*-(2-chlorophenyl)pent-4-enamide **(5l)**

MS (ESI-TOF) *m/z* calcd For CHNO (M + H)$^+$: 291.10, Found: 291.12; % yield: 85%;

4 NMR Spectra of 3a & 5(a-g)

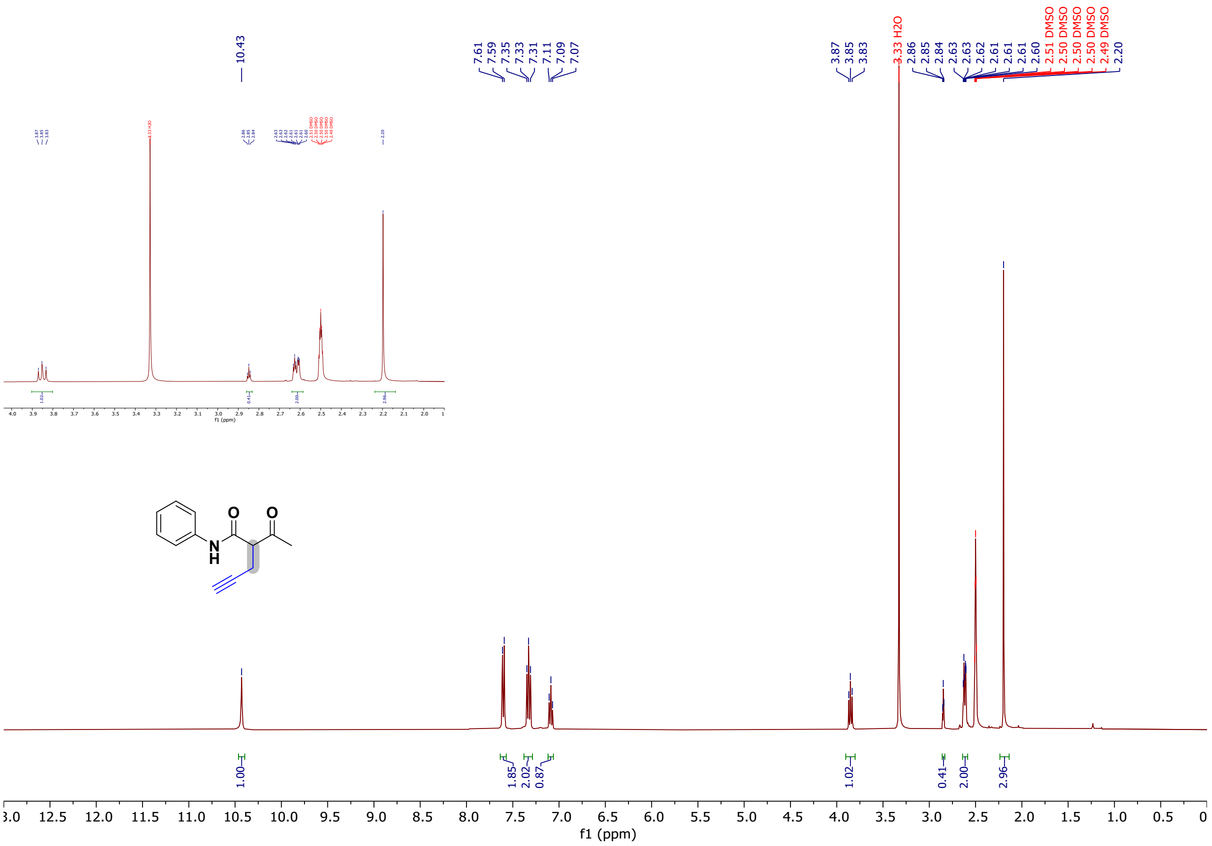

Figure S1: ^{1}H NMR spectra of compound **3a** (400 MHz, DMSO-d_6)

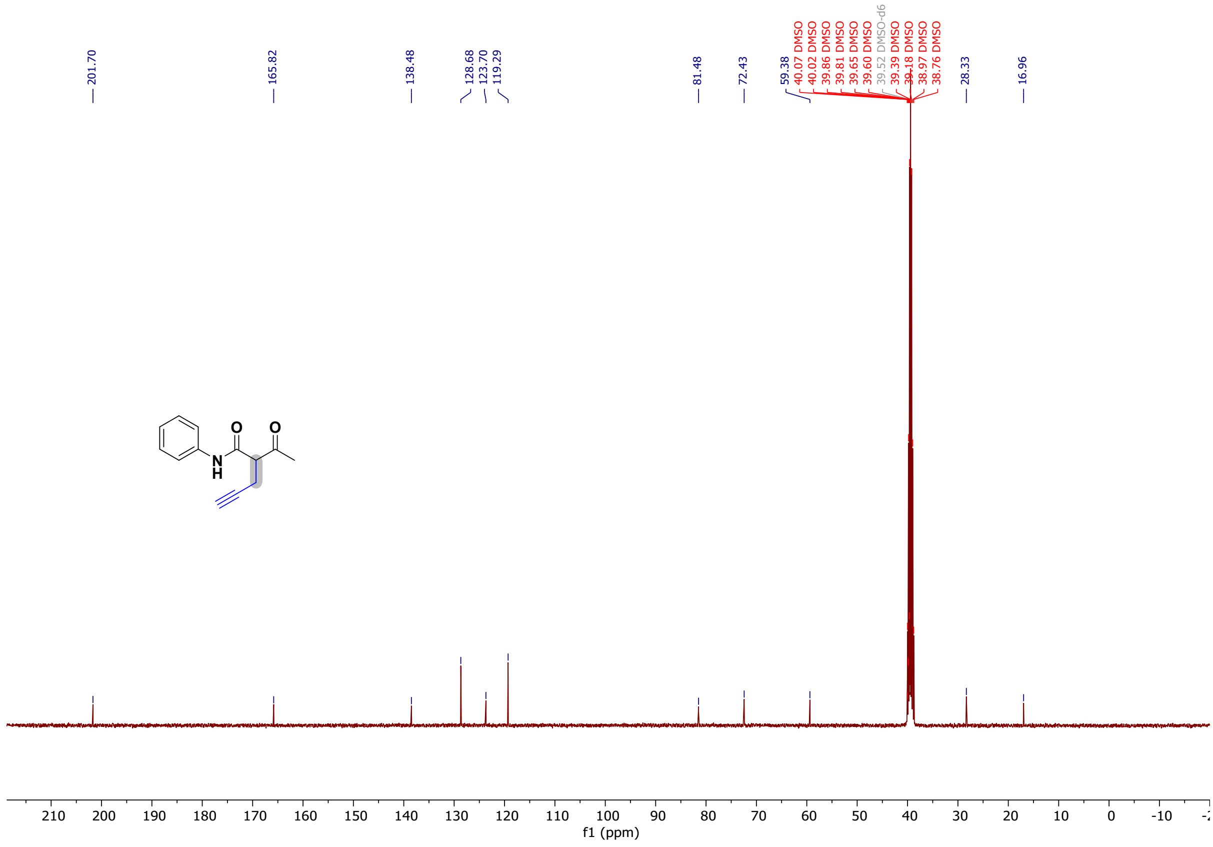

Figure S2: ^{13}C NMR spectra of compound **3a** (101 MHz, DMSO-d_6)

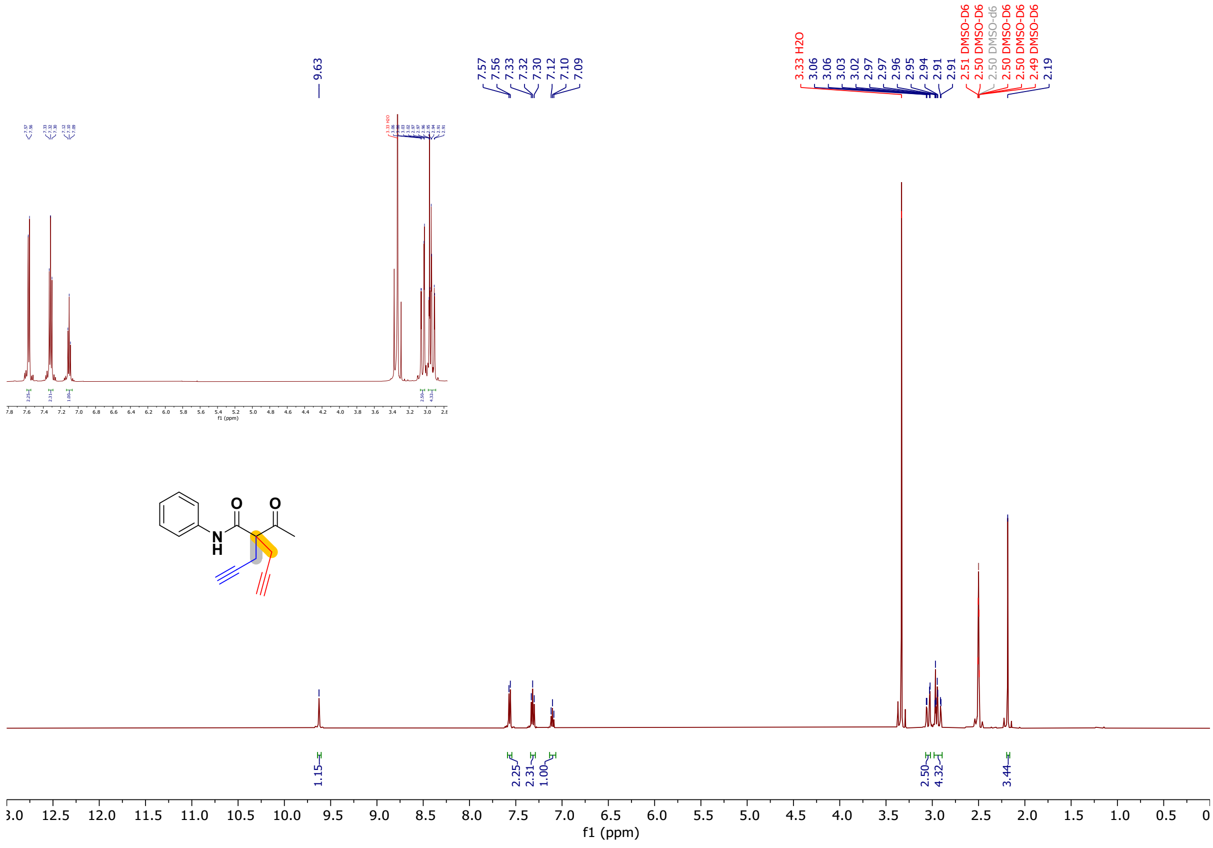

Figure S3: ^{1}H NMR spectra of **5a** (500 MHz, DMSO-d_6)

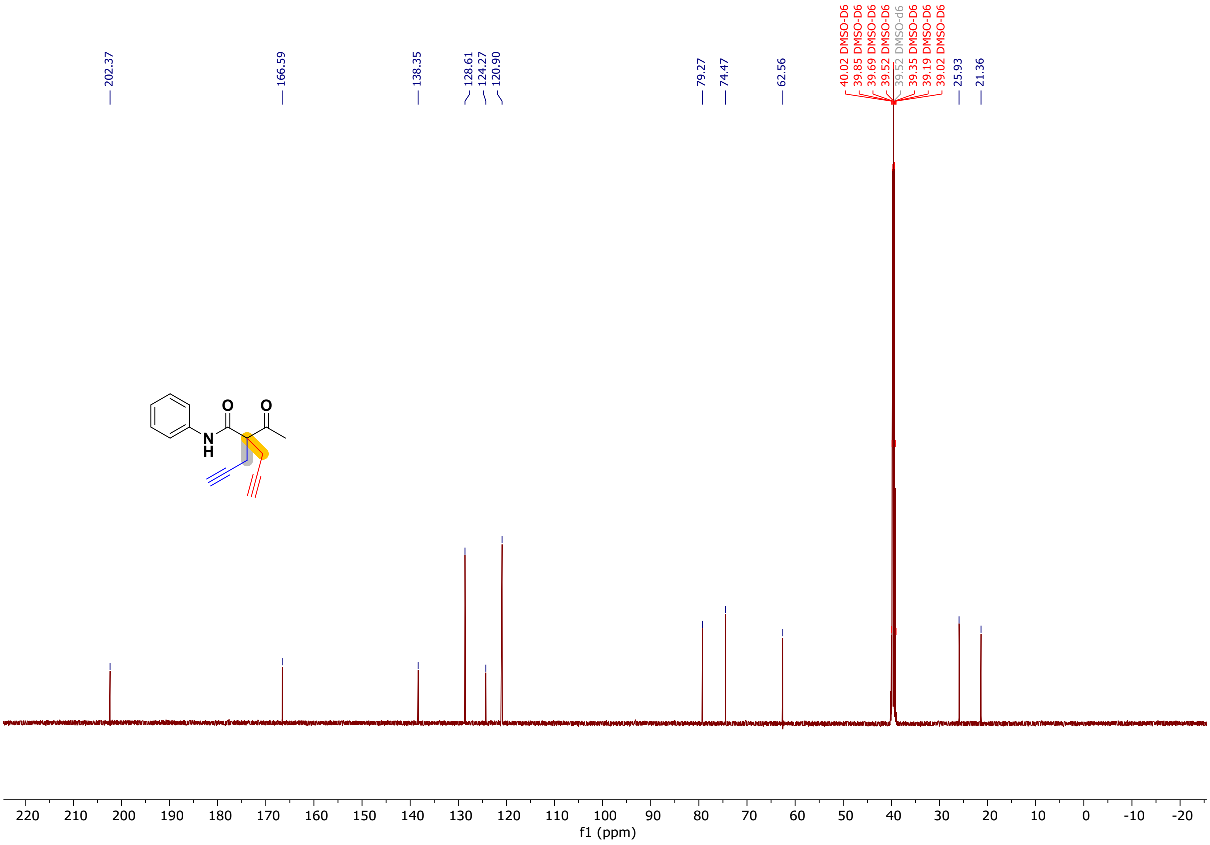

Figure S4: ^{13}C NMR spectra of **5a** (126 MHz, DMSO-d_6)

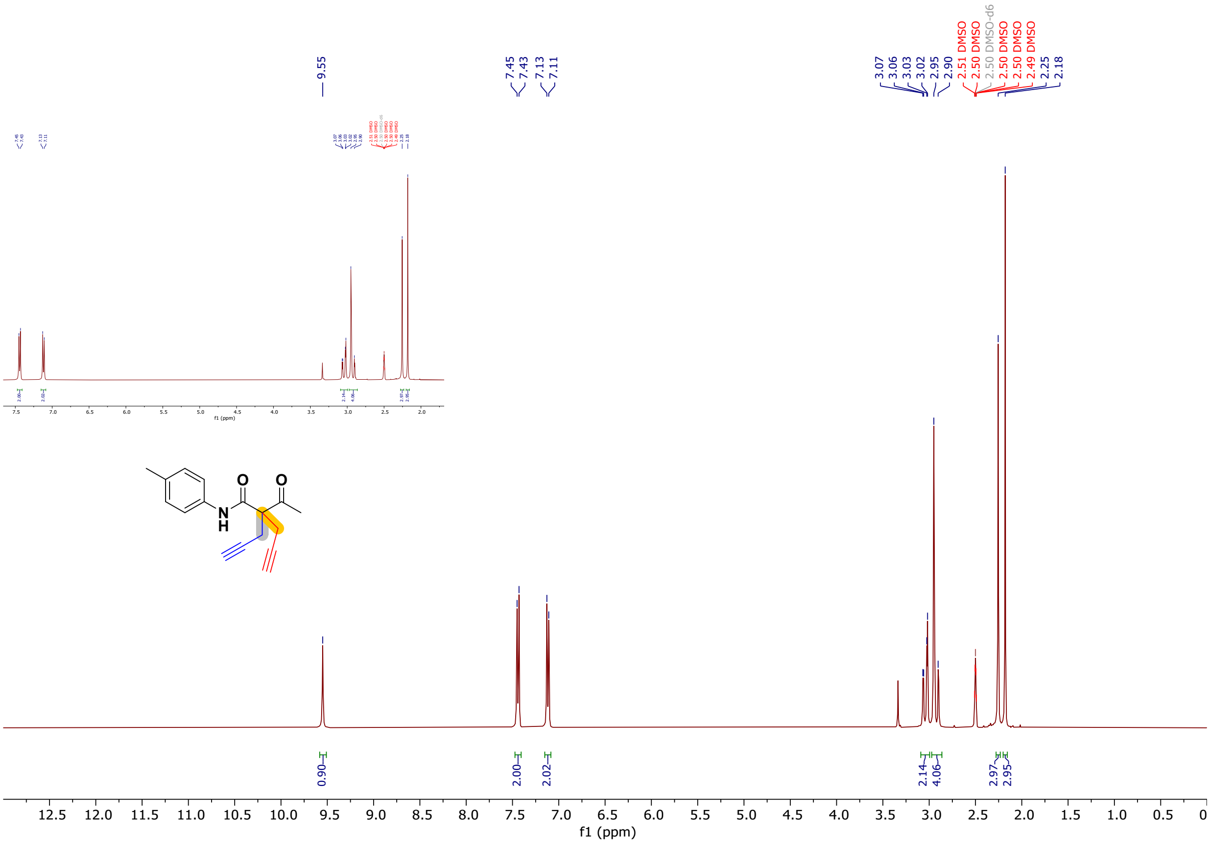

Figure S5: ^{1}H NMR spectra of **5b** (400 MHz, DMSO-d_6)

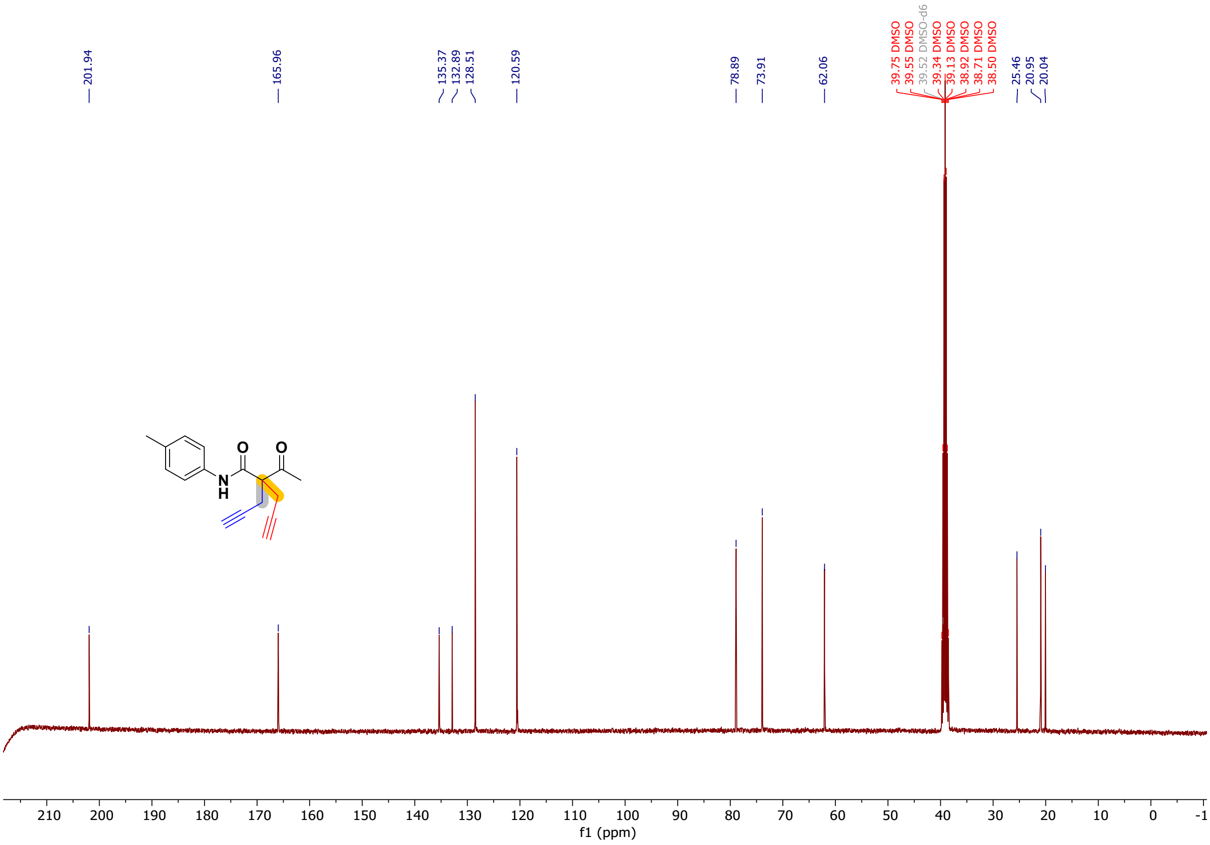

Figure S6: ^{13}C NMR spectra of **5b** (101 MHz, DMSO-d_6)

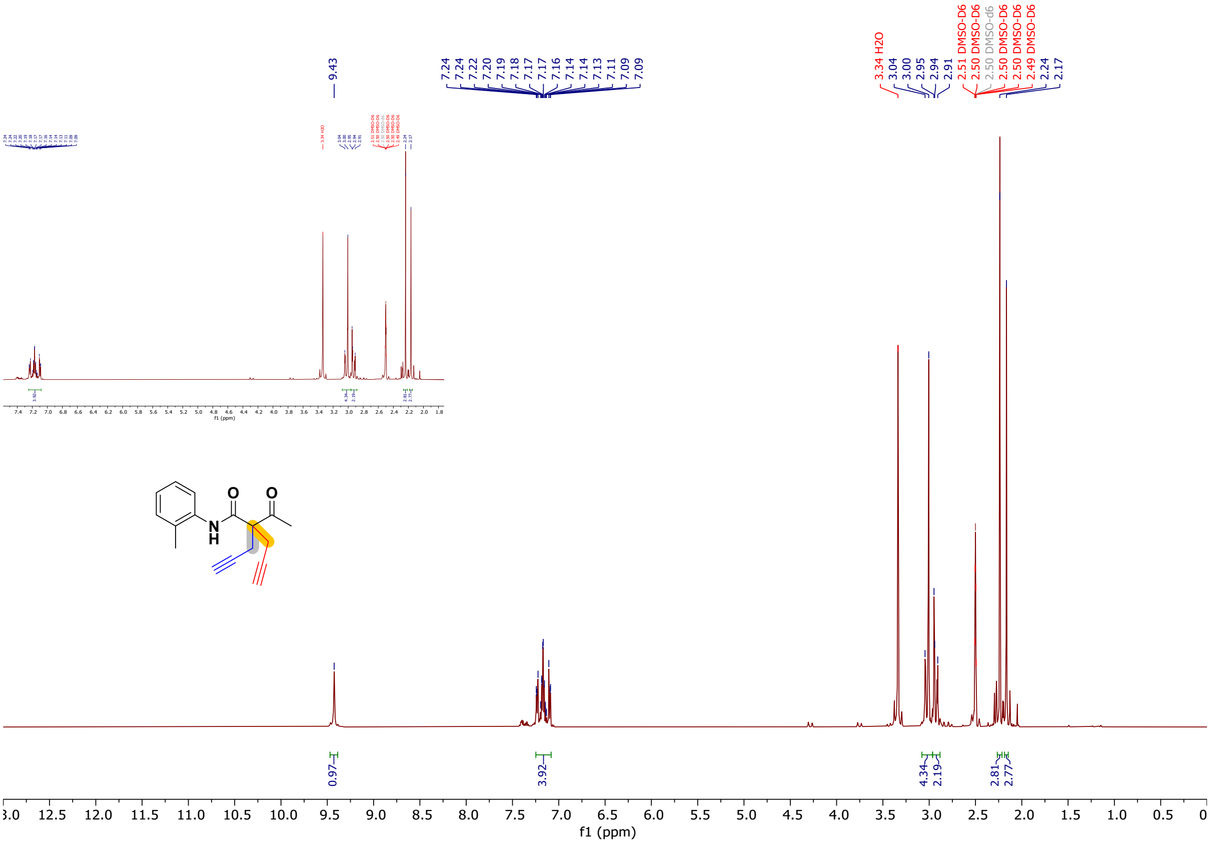

Figure S7: ^{1}H NMR spectra of **5c** (500 MHz, DMSO-d_6)

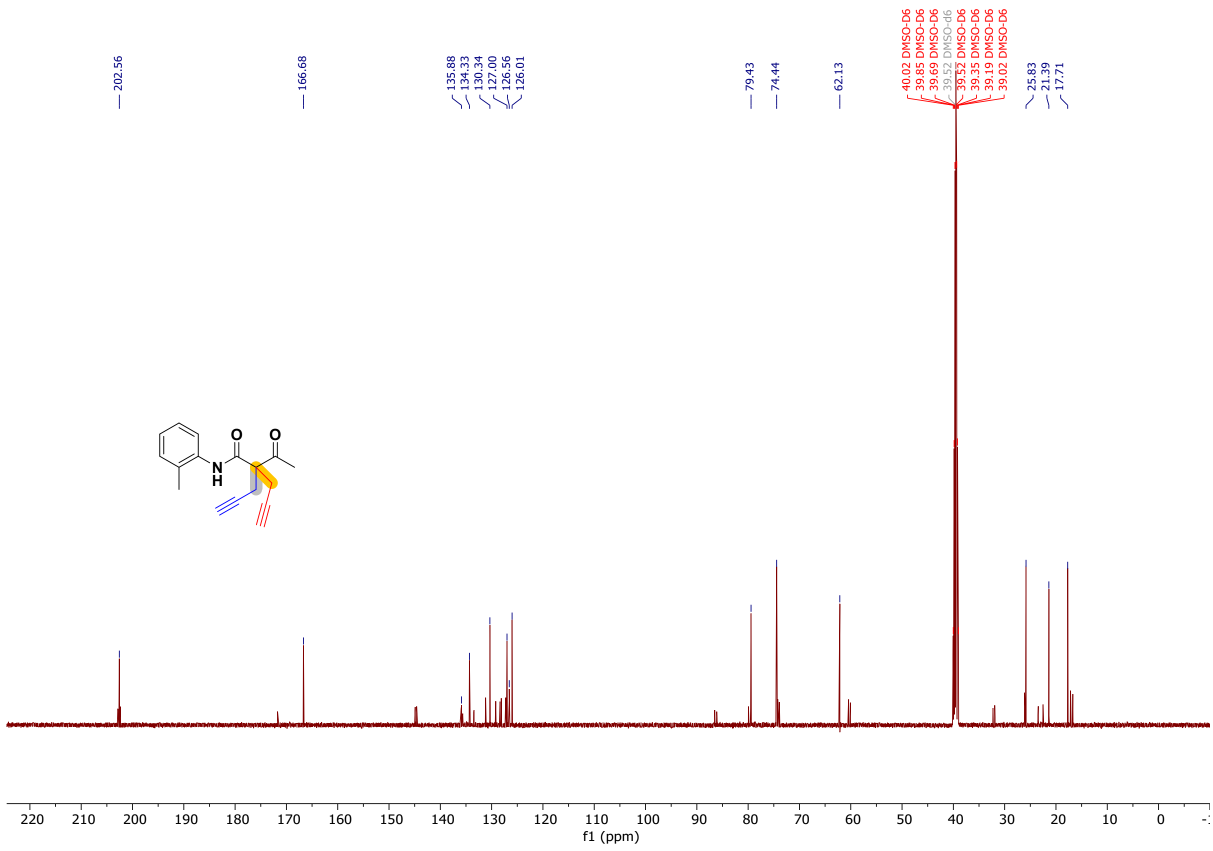

Figure S8: ^{13}C NMR spectra of **5c** (126 MHz, DMSO-d_6)

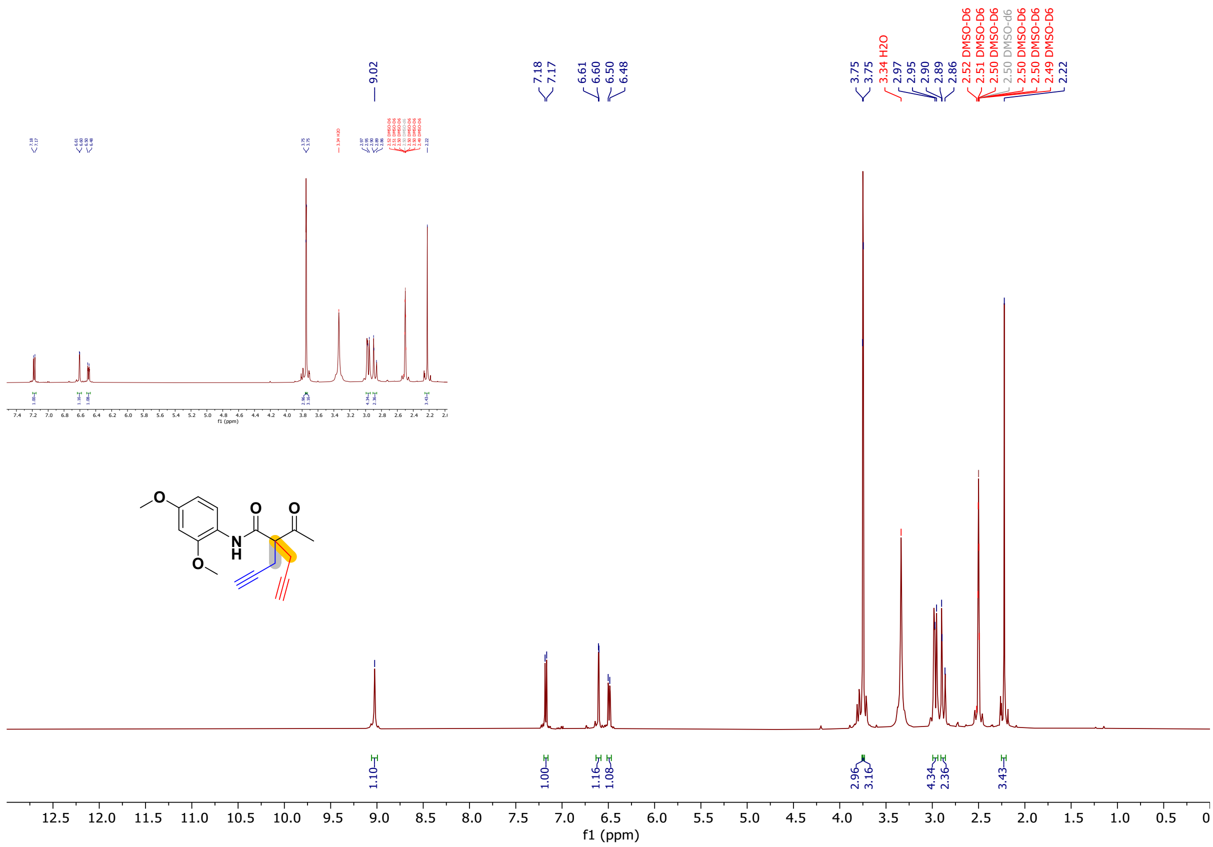

Figure S9: ^{1}H NMR spectra of **5f** (500 MHz, DMSO-d_6)

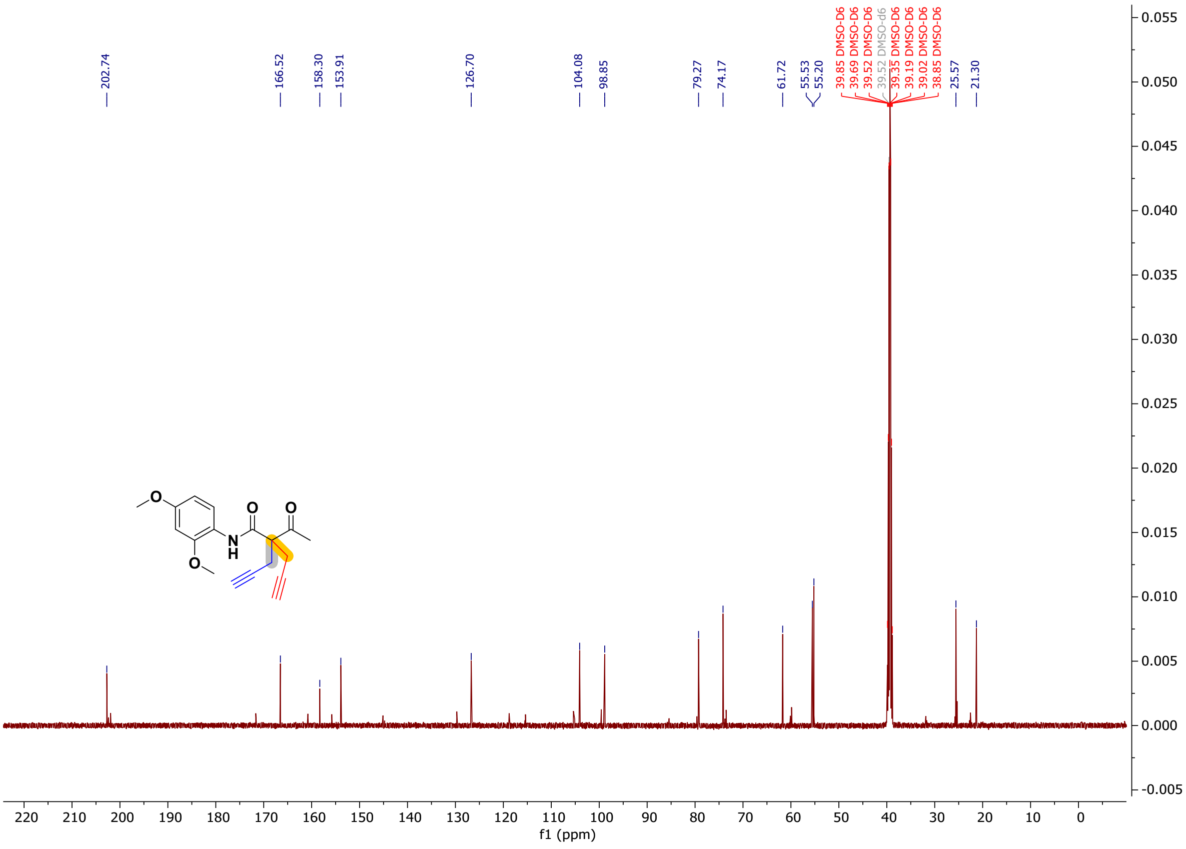

Figure S10: ^{13}C NMR spectra of **5f** (126 MHz, DMSO-d_6)

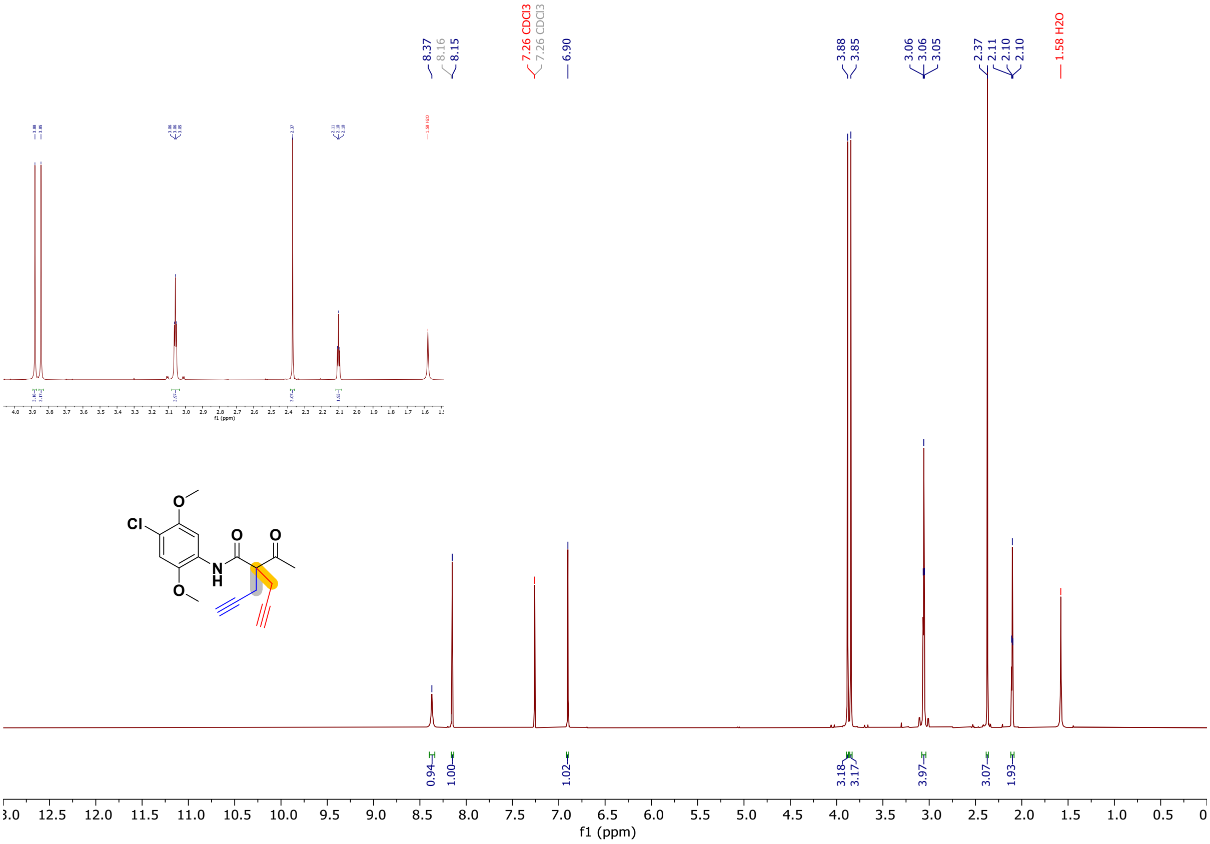

Figure S11: ^{1}H NMR spectra of **5g** (400 MHz, $CDCl_3$)

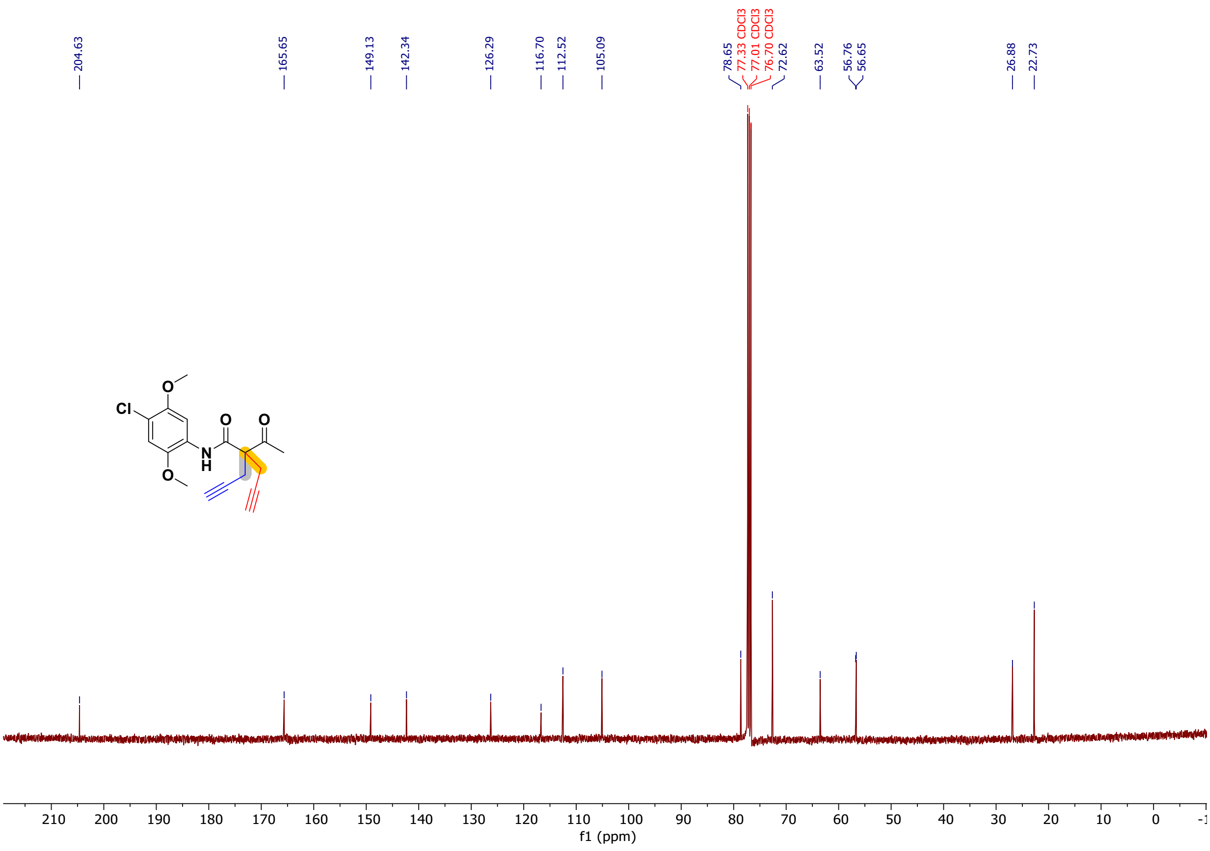

Figure S12: ^{13}C NMR spectra of **5g** (101 MHz, $CDCl_3$)

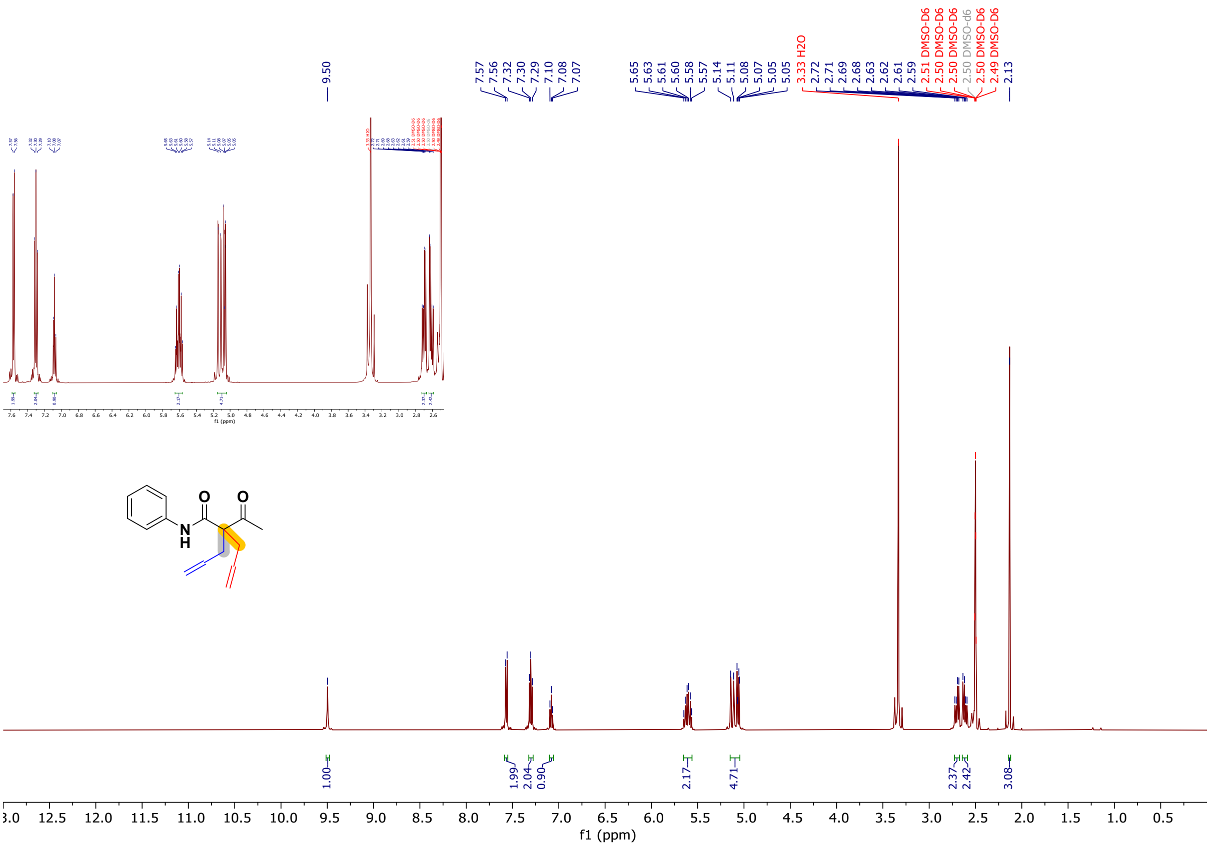

Figure S13: ^{1}H NMR spectra of **5h** (500 MHz, DMSO-d_6)

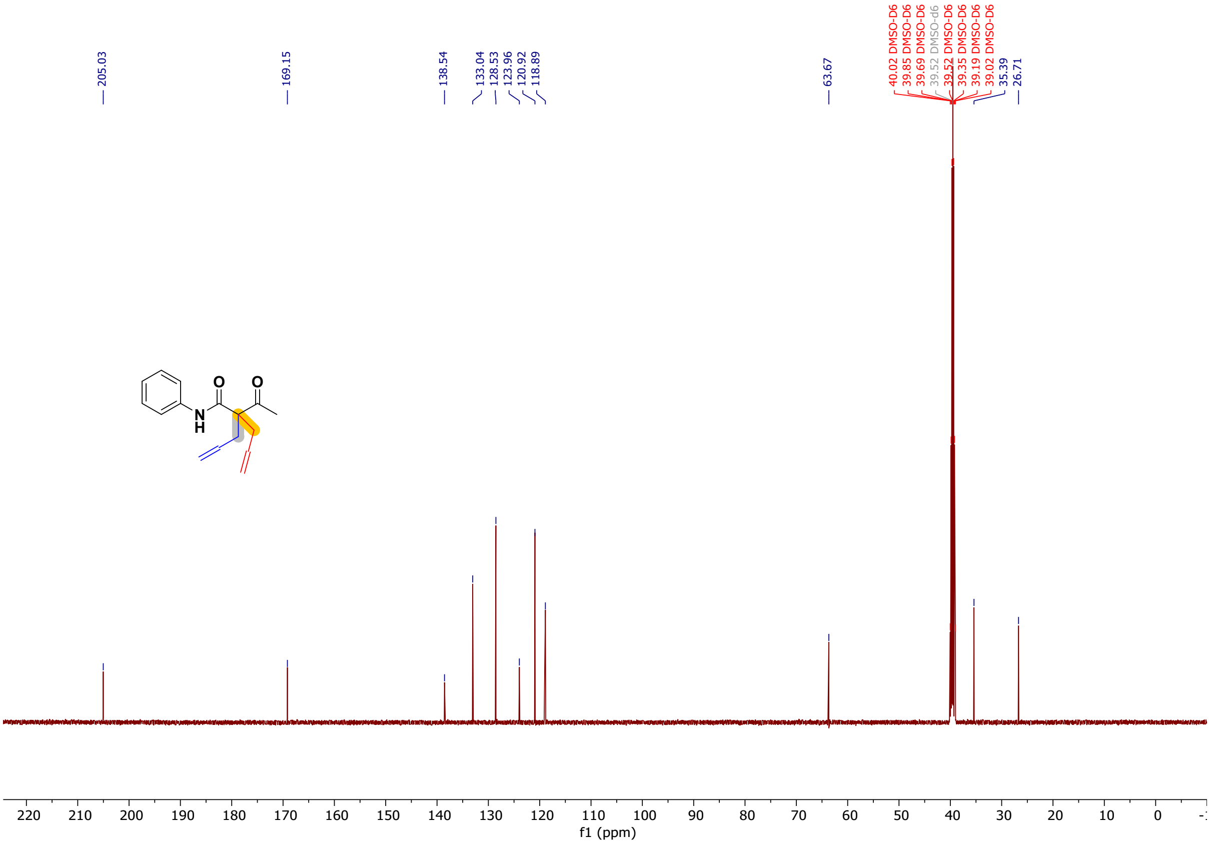

Figure S14: ^{13}C NMR spectra of **5h** (126 MHz, DMSO-d_6)

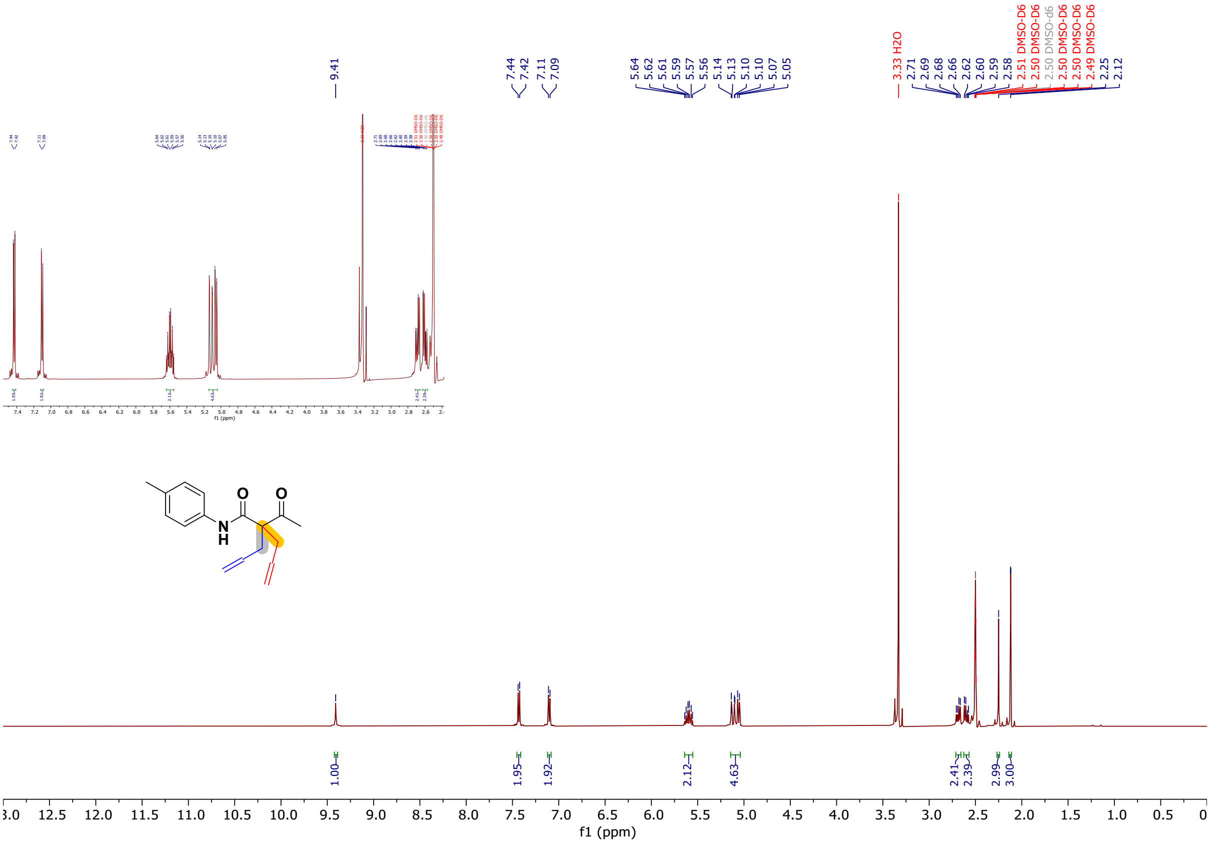

Figure S15: ^{1}H NMR spectra of **5i** (500 MHz, DMSO-d_6)

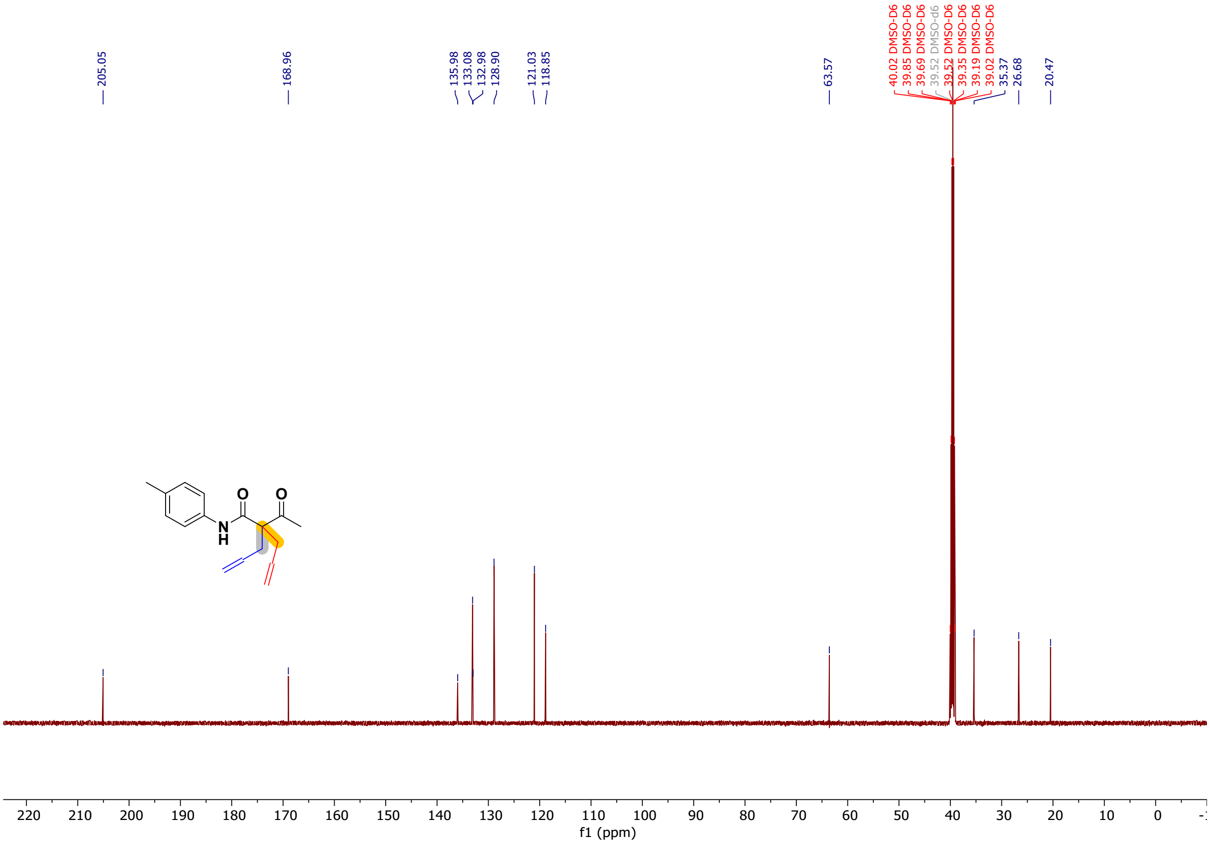

Figure S16: ^{13}C NMR spectra of **5i** (126 MHz, DMSO-d_6)

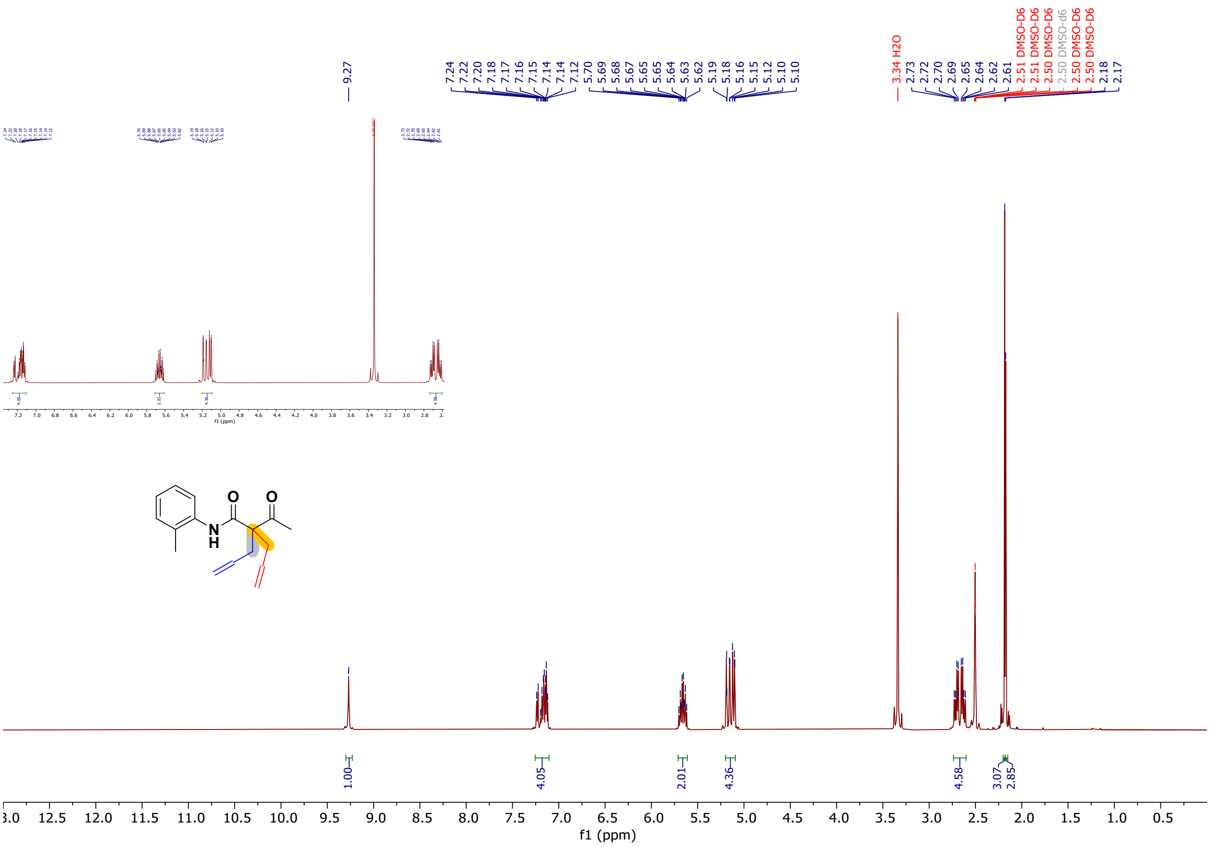

Figure S17: ^{1}H NMR spectra of **5j** (500 MHz, DMSO-d_6)

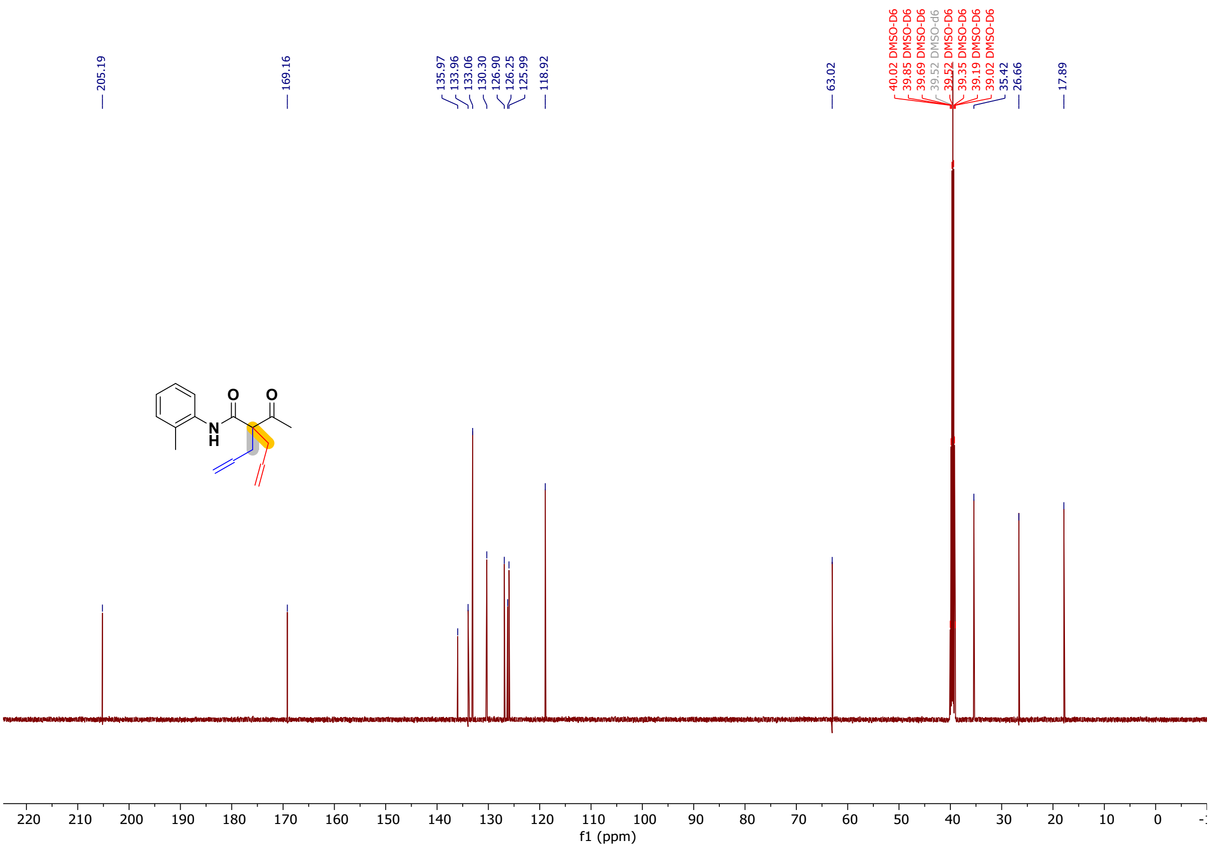

Figure S18: ^{13}C NMR spectra of **5j** (126 MHz, DMSO-d_6)

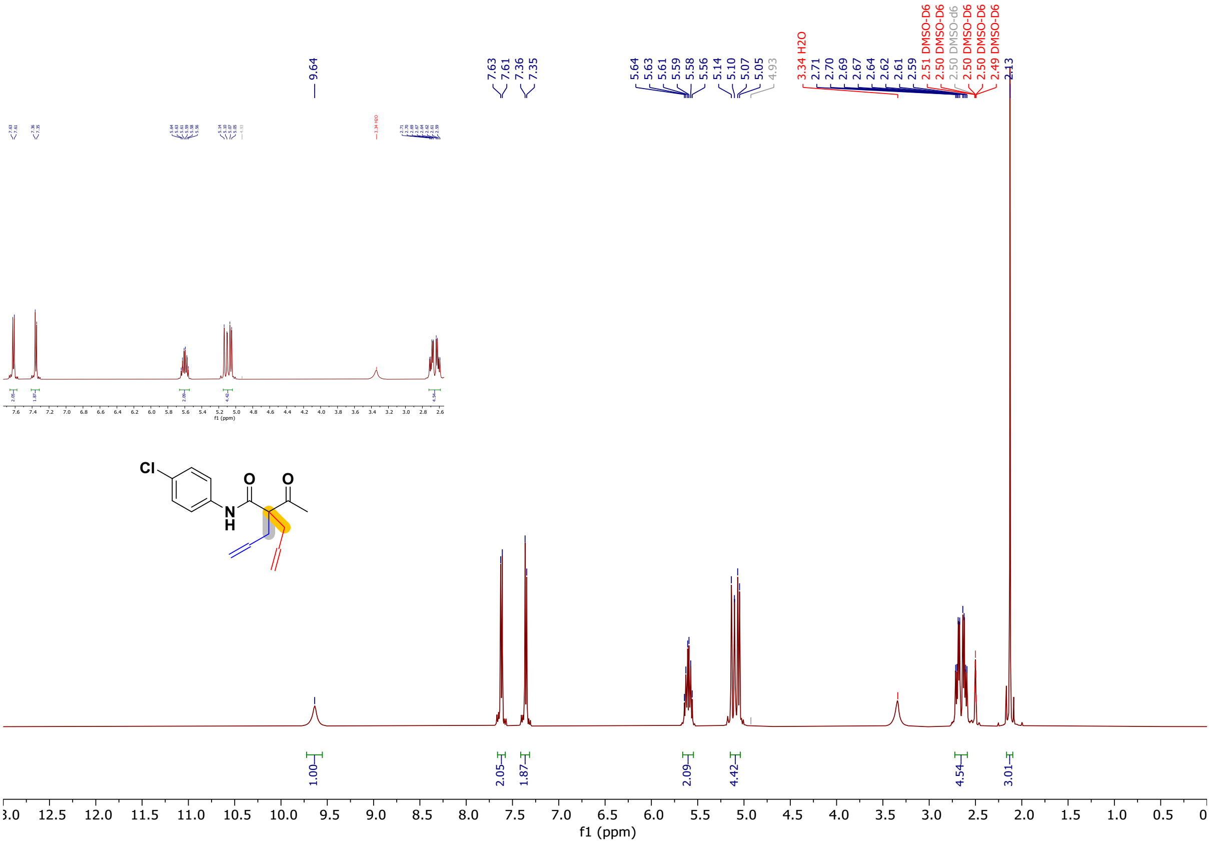

Figure S19: ^{1}H NMR spectra of **5k** (500 MHz, DMSO-d_6)

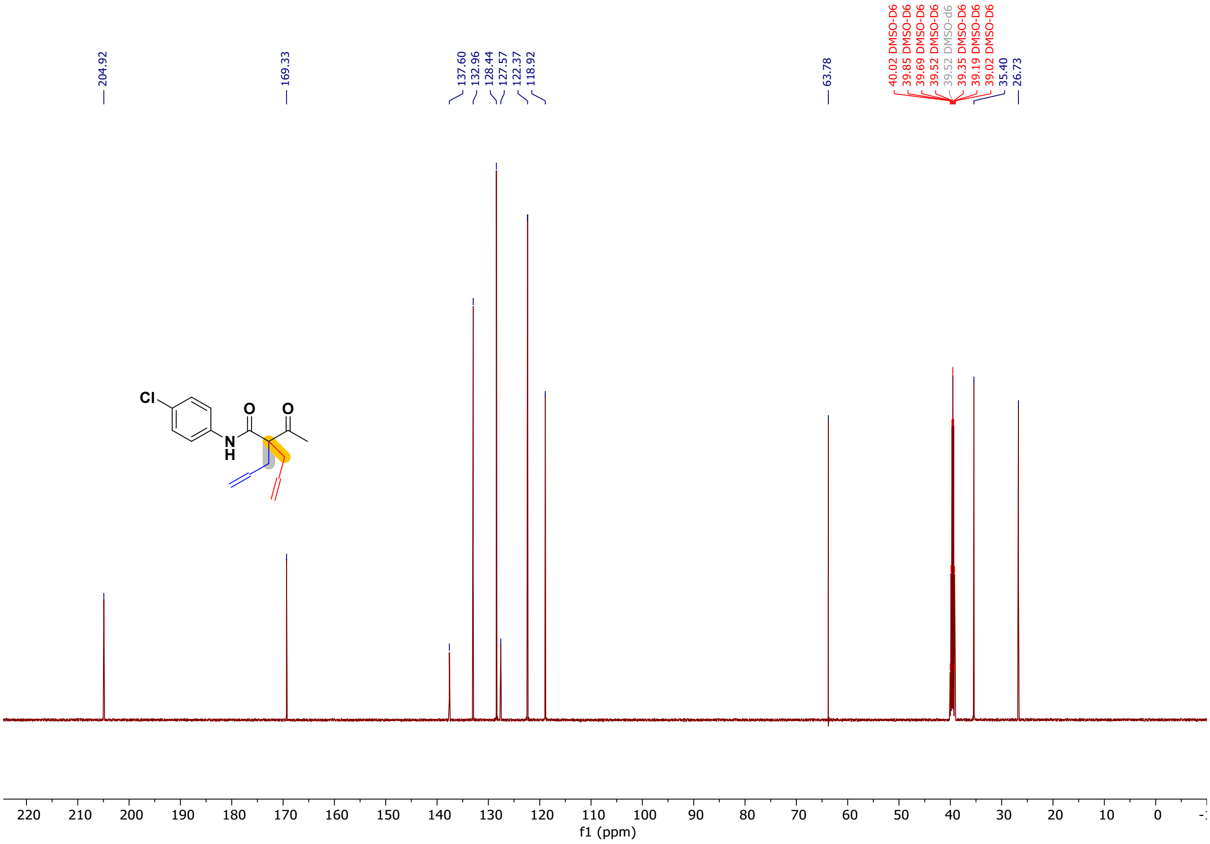

Figure S20: ^{13}C NMR spectra of **5k** (126 MHz, DMSO-d_6)

5 Mass Analysis image

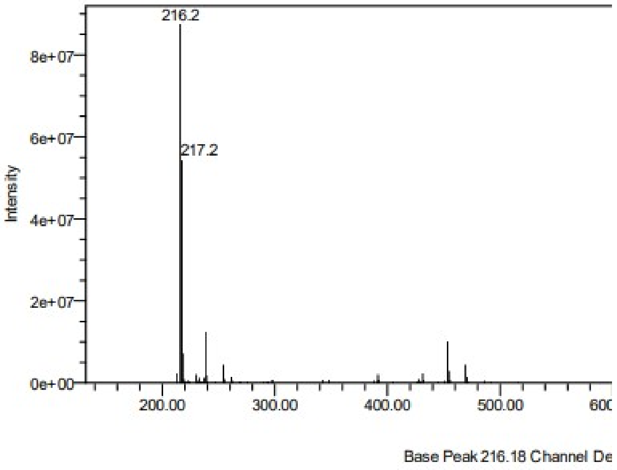

Figure S21: MS (ESI-TOF) *m/z* of **3a**

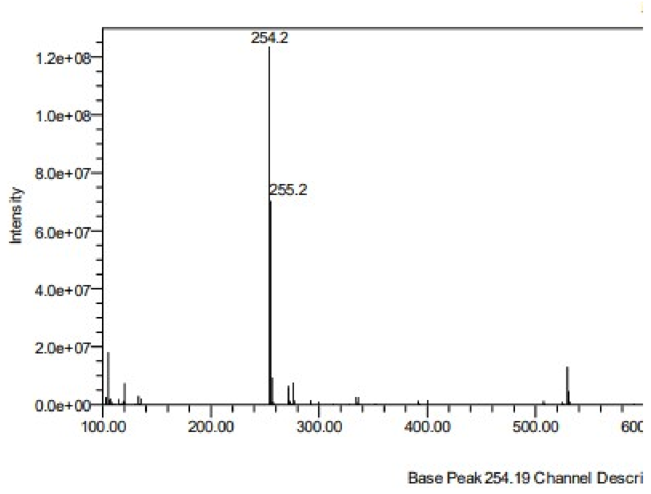

Figure S22: MS (ESI-TOF) *m/z* of **5a**

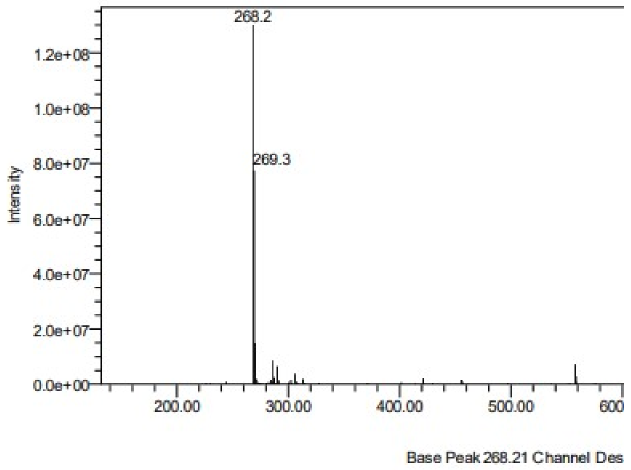

Figure S23: MS (ESI-TOF) *m/z* of **5b**

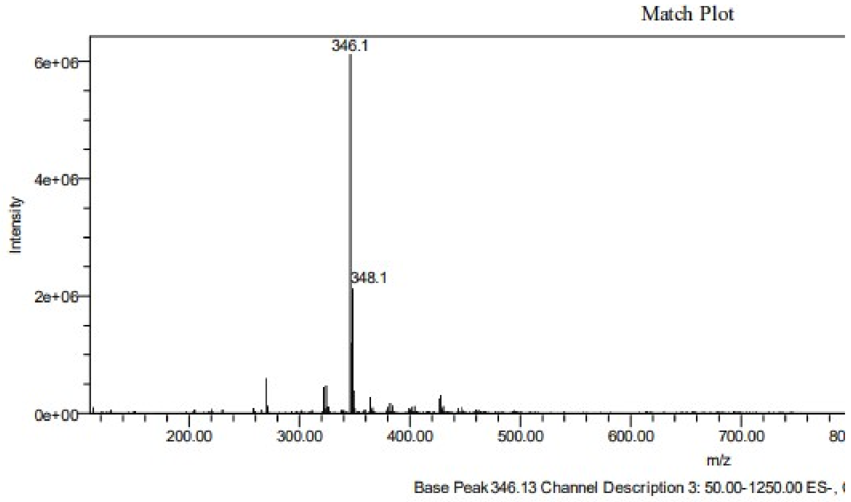

Figure S24: MS (ESI-TOF) *m/z* of **5g**

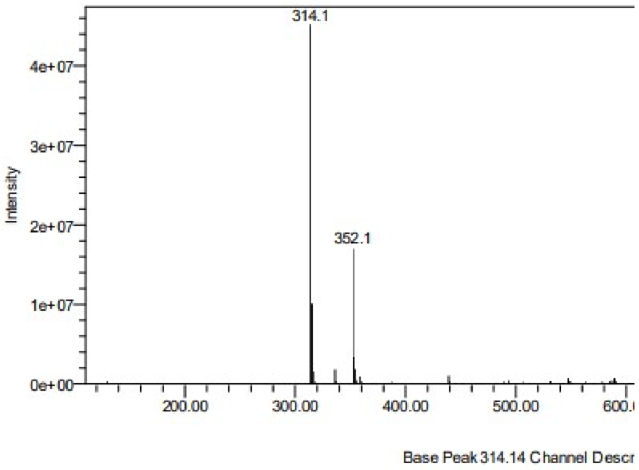

Figure S25: MS (ESI-TOF) *m/z* of **5h**

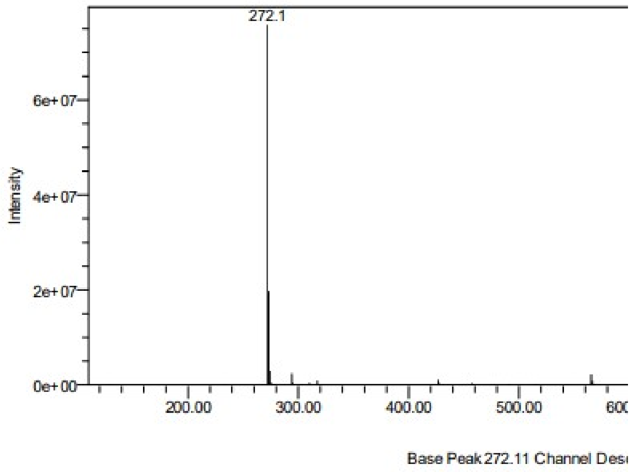

Figure S26: MS (ESI-TOF) *m/z* of **5i**

6 Crystallographic investigation

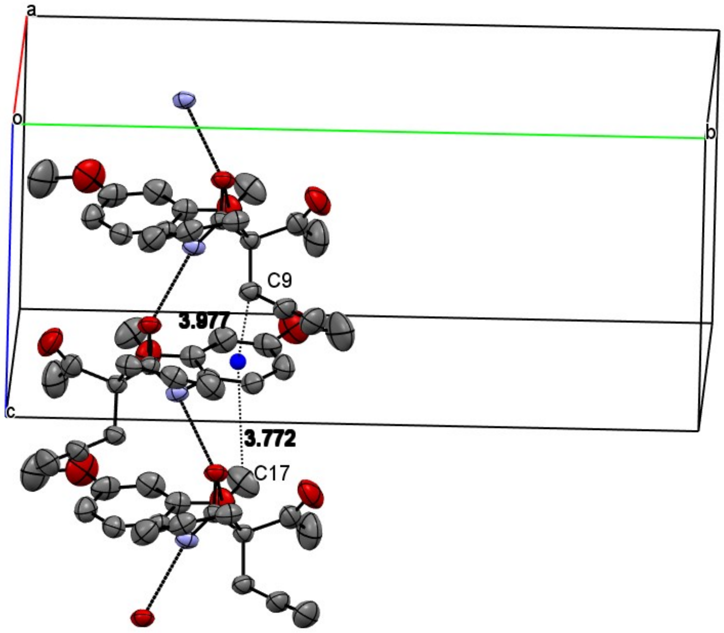

Figure S27: intermolecular sandwich type C-H…π internation

Crystal description	Colourless	
Crystal Size	0.080 x 0.160 x 0.290 mm	
Empirical formula	$C_{18}H_{19}NO_4$	
Formula weight	313.34	
Radiation, Wavelength	Mo *K*α, 0.71073 Å	
Unit cell dimension	a = 10.3767(5) Å	α = 90°
	b = 20.8762(10) Å	β = 113.4574(19)°
	c = 8.8540(3) Å	ɣ = 90°
Crystal system	Monoclinic	
Space group	p-1 21/c 1	
Unit cell volume	1759.50(14) $Å^3$	
No. of molecule per unit cell, Z	4	
Temperature	296(2) K	
Absorption coefficient	0.084 mm^{-1}	
F(000)	644	
Method of absorption correction	Multi-scan	
θ range for the entire data collection	1.95 to 25.00°	
Density (calculated)	1.183 g/cm^3	

Table S1: Preliminary Crystal data and structure refinement for **5f**

7 DFT Calculations

7.1 Optimization

The DFT studies were carried out using the B3LYP method with the 6-311G(d,p) basis set. The optimization of molecular structures using DFT showed excellent convergence for most of the systems, with all key parameters well within the defined thresholds. The mean maximum force across the systems was approximately 0.00003, significantly below the threshold of 0.000450. The RMS force also averaged at around 0.00052, comfortably meeting the threshold of 0.000300. For displacements, the mean maximum displacement was calculated at 0.00102, well within the allowable limit of 0.001800, and the mean RMS displacement was 0.00067, which also satisfied the threshold of 0.001200. These results indicate successful structural optimization for the majority of systems, with the exception of a few intermediates (INT-IIa, INT-IIb, INT-IIIb) and a transition state (TS-III) where slight deviations in force and displacement values were noted. Despite these exceptions, the overall convergence performance was robust and in line with the expectations for accurate molecular structure optimization.

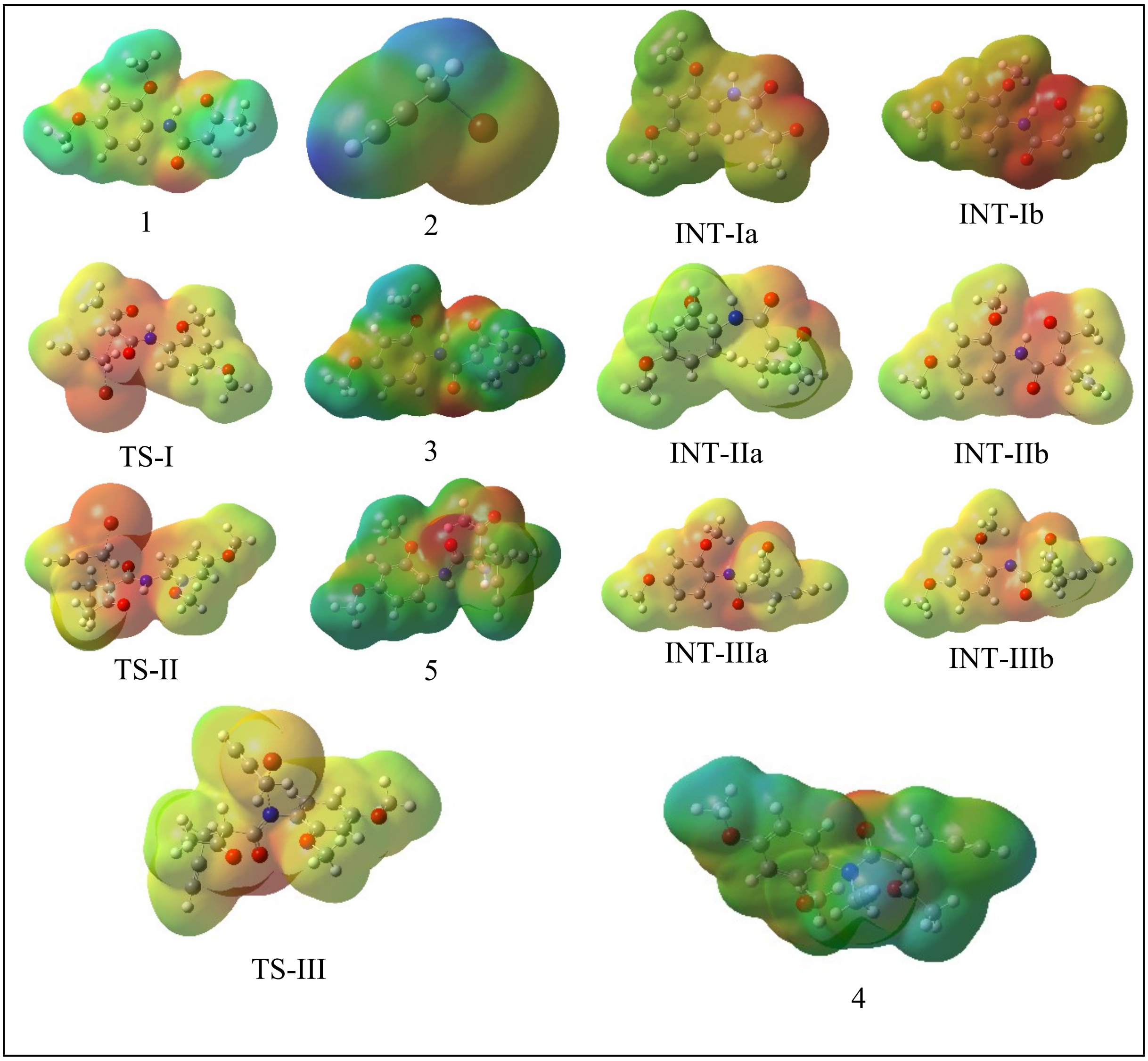

Figure S28. Electrostatic potential surface of all molecule

	H (eV)	L (eV)	(ΔE) (eV)	χ (eV)	η (eV)	s (eV^{-1})	μ (eV)	ω(eV)	ΔN max
1a	-5.22	-1.16	4.06	3.19	2.03	0.49	−3.19	2.50	1.57
INT Ia	-5.79	-0.94	4.84	3.36	2.42	0.41	−3.36	2.34	1.39
INT Ib	-4.58	-0.06	4.52	2.32	2.26	0.44	-2.32	1.19	1.02
2	-7.56	-1.38	6.17	4.47	3.08	0.32	-4.47	3.24	1.45
TS-I	-5.29	-2.58	2.71	3.94	1.35	0.73	-3.94	5.71	2.90
3	-5.61	-1.37	4.24	3.49	2.12	0.47	-3.49	2.87	1.64
INT IIIa	-4.57	-0.30	4.27	2.43	2.13	0.46	−2.43	1.39	1.14
INT IIIb	-4.58	-0.64	3.93	2.61	1.96	0.50	-2.61	1.73	1.32
INT IIa	-4.48	-0.01	4.47	2.25	2.23	0.44	-2.25	1.13	1.00
INT IIb	-4.51	-0.05	4.45	2.28	2.22	0.44	-2.28	1.16	1.02
TS-II	-5.28	-2.63	2.65	3.96	1.82	0.54	−3.96	7.23	2.17
TS-III	-5.41	-2.23	3.18	3.82	1.59	0.62	−3.82	4.59	2.40
**4**	**-6.16**	**-1.32**	**4.84**	**3.74**	**2.42**	**0.41**	**−3.74**	**5.52**	**1.54**
**5**	**-6.10**	**-1.12**	**4.98**	**3.61**	**2.49**	**0.40**	**−3.61**	**5.14**	**1.45**
	HOMO(H), LUMO(L), Energy Gap (ΔE), Electronegativity (χ), Chemical Hardness (η), Chemical Softness (s), Chemical Potential (μ), Electrophilicity (ω), ΔN max								

Table S2: Quantum Chemical calculation

**1**			
Item	Value	Threshold	Converged?
Maximum Force	0.000009	0.000450	YES
RMS Force	0.000002	0.000300	YES
Maximum Displacement	0.001187	0.001800	YES
RMS Displacement	0.000200	0.001200	YES
**2**			
Item	Value	Threshold	Converged?
Maximum Force	0.000155	0.000450	YES
RMS Force	0.000058	0.000300	YES
Maximum Displacement	0.001106	0.001800	YES
RMS Displacement	0.000410	0.001200	YES
**INT-Ia**			
Item	Value	Threshold	Converged?
Item	0.000037	0.000450	YES
Maximum Force	0.000008	0.000300	YES
RMS Force	0.002716	0.001800	YES
Maximum Displacement	0.000590	0.001200	YES
**INT-Ib**			
Item	Value	Threshold	Converged?
Item	0.000002	0.000450	YES
Maximum Force	0.000001	0.000300	YES
RMS Force	0.000901	0.001800	YES
Maximum Displacement	0.000140	0.001200	YES
**TS-I**			
Item	Value	Threshold	Converged?
Item	0.000002	0.000450	YES
Maximum Force	0.000000	0.000300	YES

RMS Force	0.000982	0.001800	YES
Maximum Displacement	0.000193	0.001200	YES
**3**			
Item	Value	Threshold	Converged?
Item	0.000005	0.000450	YES
Maximum Force	0.000001	0.000300	YES
RMS Force	0.001858	0.001800	YES
Maximum Displacement	0.000522	0.001200	YES
**INT-IIa**			
Item	Value	Threshold	Converged?
Item	0.000013	0.000450	YES
Maximum Force	0.000003	0.000300	YES
RMS Force	0.002840	0.001800	NO
Maximum Displacement	0.000577	0.001200	YES
**INT-IIb**			
Item	Value	Threshold	Converged?
Item	0.000011	0.000450	YES
Maximum Force	0.000003	0.000300	YES
RMS Force	0.003145	0.001800	NO
Maximum Displacement	0.000622	0.001200	YES
**TS-II**			
Item	Value	Threshold	Converged?
Item	0.000009	0.000450	YES
Maximum Force	0.000001	0.000300	YES
RMS Force	0.001257	0.001800	YES
Maximum Displacement	0.000229	0.001200	YES
**5**			
Item	Value	Threshold	Converged?
Item	0.000051	0.000450	YES
Maximum Force	0.000009	0.000300	YES
RMS Force	0.001673	0.001800	YES
Maximum Displacement	0.000345	0.001200	YES
**INT-IIIa**			
Item	Value	Threshold	Converged?
Item	0.000015	0.000450	YES
Maximum Force	0.000003	0.000300	YES
RMS Force	0.000897	0.001800	YES
Maximum Displacement	0.000254	0.001200	YES
**INT-IIIb**			
Item	Value	Threshold	Converged?
Item	0.000058	0.000450	YES
Maximum Force	0.000012	0.000300	YES
RMS Force	0.007341	0.001800	NO
Maximum Displacement	0.001753	0.001200	NO
**TS-III**			
Item	Value	Threshold	Converged?
Item	0.000046	0.000450	YES
Maximum Force	0.000010	0.000300	YES
RMS Force	0.053944	0.001800	NO

Maximum Displacement	0.011020	0.001200	NO
**4**			
Item	Value	Threshold	Converged?
Item	0.000017	0.000450	YES
Maximum Force	0.000003	0.000300	YES
RMS Force	0.001416	0.001800	YES
Maximum Displacement	0.000242	0.001200	YES

Table S3: Optimized Parameter

1	Electronic Energy (EE) = -821.71484 Hartree<br>Zero-point Energy Correction = 0.260508 Hartree<br>Thermal Correction to Energy = 0.277801 Hartree<br>Thermal Correction to Enthalpy = 0.278745 Hartree<br>Thermal Correction to Free Energy = 0.213889 Hartree<br>EE + Zero-point Energy = -821.45433 Hartree<br>EE + Thermal Energy Correction = -821.43704 Hartree<br>EE + Thermal Enthalpy Correction = -821.43609 Hartree<br>EE + Thermal Free Energy Correction = -821.50095 Hartree<br>E (Thermal) = 174.323 kcal/mol<br>Heat Capacity (Cv) = 62.761 cal/mol-kelvin<br>Entropy (S) = 136.5 cal/mol-kelvin
2	Electronic Energy (EE) = -2687.5987 Hartree<br>Zero-point Energy Correction = 0.047557 Hartree<br>Thermal Correction to Energy = 0.05216 Hartree<br>Thermal Correction to Enthalpy = 0.053104 Hartree<br>Thermal Correction to Free Energy = 0.018942 Hartree<br>EE + Zero-point Energy = -2687.5511 Hartree<br>EE + Thermal Energy Correction = -2687.5465 Hartree<br>EE + Thermal Enthalpy Correction = -2687.5456 Hartree<br>EE + Thermal Free Energy Correction = -2687.5797 Hartree<br>E (Thermal) = 32.731 kcal/mol<br>Heat Capacity (Cv) = 14.841 cal/mol-kelvin<br>Entropy (S) = 71.9 cal/mol-kelvin
3	Electronic Energy (EE) = -937.15369 Hartree<br>Zero-point Energy Correction = 0.297954 Hartree<br>Thermal Correction to Energy = 0.318605 Hartree<br>Thermal Correction to Enthalpy = 0.319549 Hartree<br>Thermal Correction to Free Energy = 0.246829 Hartree<br>EE + Zero-point Energy = -936.85574 Hartree<br>EE + Thermal Energy Correction = -936.83509 Hartree<br>EE + Thermal Enthalpy Correction = -936.83414 Hartree<br>EE + Thermal Free Energy Correction = -936.90686 Hartree<br>E (Thermal) = 199.928 kcal/mol<br>Heat Capacity (Cv) = 75.491 cal/mol-kelvin<br>Entropy (S) = 153.051 cal/mol-kelvin
4	Electronic Energy (EE) = -1052.5493 Hartree<br>Zero-point Energy Correction = 0.333979 Hartree<br>Thermal Correction to Energy = 0.358543 Hartree

	Thermal Correction to Enthalpy = 0.359487 Hartree<br>Thermal Correction to Free Energy = 0.276935 Hartree<br>EE + Zero-point Energy = -1052.2153 Hartree<br>EE + Thermal Energy Correction = -1052.1908 Hartree<br>EE + Thermal Enthalpy Correction = -1052.1898 Hartree<br>EE + Thermal Free Energy Correction = -1052.2724 Hartree<br>E (Thermal) = 224.989 kcal/mol<br>Heat Capacity (Cv) = 88.473 cal/mol-kelvin<br>Entropy (S) = 173.745 cal/mol-kelvin
INT-Ia	Electronic Energy (EE) = -821.22944 Hartree<br>Zero-point Energy Correction = 0.246727 Hartree<br>Thermal Correction to Energy = 0.263958 Hartree<br>Thermal Correction to Enthalpy = 0.264902 Hartree<br>Thermal Correction to Free Energy = 0.200415 Hartree<br>EE + Zero-point Energy = -820.98271 Hartree<br>EE + Thermal Energy Correction = -820.96548 Hartree<br>EE + Thermal Enthalpy Correction = -820.96454 Hartree<br>EE + Thermal Free Energy Correction = -821.02903 Hartree<br>E (Thermal) = 165.636 kcal/mol<br>Heat Capacity (Cv) = 62.699 cal/mol-kelvin<br>Entropy (S) = 135.725 cal/mol-kelvin
INT-Ib	Electronic Energy (EE) = -821.24668 Hartree<br>Zero-point Energy Correction = 0.24657 Hartree<br>Thermal Correction to Energy = 0.263605 Hartree<br>Thermal Correction to Enthalpy = 0.264549 Hartree<br>Thermal Correction to Free Energy = 0.200837 Hartree<br>EE + Zero-point Energy = -821.00011 Hartree<br>EE + Thermal Energy Correction = -820.98307 Hartree<br>EE + Thermal Enthalpy Correction = -820.98213 Hartree<br>EE + Thermal Free Energy Correction = -821.04584 Hartree<br>E (Thermal) = 165.415 kcal/mol<br>Heat Capacity (Cv) = 62.062 cal/mol-kelvin<br>Entropy (S) = 134.095 cal/mol-kelvin
TS-I	Zero-point correction= 0.290242 (Hartree/Particle)<br>Thermal correction to Energy= 0.313748<br>Thermal correction to Enthalpy= 0.314692<br>Thermal correction to Gibbs Free Energy= 0.232499<br>Sum of electronic and zero-point Energies= -3511.665052<br>Sum of electronic and thermal Energies= -3511.641546<br>Sum of electronic and thermal Enthalpies= -3511.640602<br>Sum of electronic and thermal Free Energies= -3511.722794
INT-IIa	Electronic Energy (EE) = -936.64053 Hartree<br>Zero-point Energy Correction = 0.283654 Hartree<br>Thermal Correction to Energy = 0.304245 Hartree<br>Thermal Correction to Enthalpy = 0.305189 Hartree<br>Thermal Correction to Free Energy = 0.232805 Hartree<br>EE + Zero-point Energy = -936.35687 Hartree<br>EE + Thermal Energy Correction = -936.33628 Hartree<br>EE + Thermal Enthalpy Correction = -936.33534 Hartree<br>EE + Thermal Free Energy Correction = -936.40772 Hartree

	E (Thermal) = 190.916 kcal/mol<br>Heat Capacity (Cv) = 75.507 cal/mol-kelvin<br>Entropy (S) = 152.344 cal/mol-kelvin
INT-IIb	Electronic Energy (EE) = -936.67202 Hartree<br>Zero-point Energy Correction = 0.283775 Hartree<br>Thermal Correction to Energy = 0.304345 Hartree<br>Thermal Correction to Enthalpy = 0.305289 Hartree<br>Thermal Correction to Free Energy = 0.232767 Hartree<br>EE + Zero-point Energy = -936.38824 Hartree<br>EE + Thermal Energy Correction = -936.36767 Hartree<br>EE + Thermal Enthalpy Correction = -936.36673 Hartree<br>EE + Thermal Free Energy Correction = -936.43925 Hartree<br>E (Thermal) = 190.979 kcal/mol<br>Heat Capacity (Cv) = 74.873 cal/mol-kelvin<br>Entropy (S) = 152.635 cal/mol-kelvin
**TS-II**	Zero-point correction= 0.327653 (Hartree/Particle)<br>Thermal correction to Energy= 0.354388<br>Thermal correction to Enthalpy= 0.355333<br>Thermal correction to Gibbs Free Energy= 0.266780<br>Sum of electronic and zero-point Energies= -3627.110175<br>Sum of electronic and thermal Energies= -3627.083440<br>Sum of electronic and thermal Enthalpies= -3627.082496<br>Sum of electronic and thermal Free Energies= -3627.171049
**5**	Zero-point correction= 0.290242 (Hartree/Particle)<br>Thermal correction to Energy= 0.313748<br>Thermal correction to Enthalpy= 0.314692<br>Thermal correction to Gibbs Free Energy= 0.232499<br>Sum of electronic and zero-point Energies= -3511.665052<br>Sum of electronic and thermal Energies= -3511.641546<br>Sum of electronic and thermal Enthalpies= -3511.640602<br>Sum of electronic and thermal Free Energies= -3511.722794
**INT-IIIa**	Electronic Energy (EE) = -936.64554 Hartree<br>Zero-point Energy Correction = 0.283052 Hartree<br>Thermal Correction to Energy = 0.30364 Hartree<br>Thermal Correction to Enthalpy = 0.304584 Hartree<br>Thermal Correction to Free Energy = 0.232445 Hartree<br>EE + Zero-point Energy = -936.36249 Hartree<br>EE + Thermal Energy Correction = -936.3419 Hartree<br>EE + Thermal Enthalpy Correction = -936.34096 Hartree<br>EE + Thermal Free Energy Correction = -936.41309 Hartree<br>E (Thermal) = 190.537 kcal/mol<br>Heat Capacity (Cv) = 75.079 cal/mol-kelvin<br>Entropy (S) = 151.829 cal/mol-kelvin
**INT-IIIb**	Electronic Energy (EE) = -936.64521 Hartree<br>Zero-point Energy Correction = 0.283016 Hartree<br>Thermal Correction to Energy = 0.303649 Hartree<br>Thermal Correction to Enthalpy = 0.304594 Hartree<br>Thermal Correction to Free Energy = 0.231807 Hartree<br>EE + Zero-point Energy = -936.36219 Hartree<br>EE + Thermal Energy Correction = -936.34156 Hartree

	EE + Thermal Enthalpy Correction = -936.34062 Hartree<br>EE + Thermal Free Energy Correction = -936.4134 Hartree<br>E (Thermal) = 190.543 kcal/mol<br>Heat Capacity (Cv) = 75.061 cal/mol-kelvin<br>Entropy (S) = 153.191 cal/mol-kelvin
**TS-III**	Electronic Energy (EE) = -3627.4138 Hartree<br>Zero-point Energy Correction = 0.326748 Hartree<br>Thermal Correction to Energy = 0.353788 Hartree<br>Thermal Correction to Enthalpy = 0.354732 Hartree<br>Thermal Correction to Free Energy = 0.264634 Hartree<br>EE + Zero-point Energy = -3627.087 Hartree<br>EE + Thermal Energy Correction = -3627.06 Hartree<br>EE + Thermal Enthalpy Correction = -3627.059 Hartree<br>EE + Thermal Free Energy Correction = -3627.1491 Hartree<br>E (Thermal) = 222.005 kcal/mol<br>Heat Capacity (Cv) = 95.377 cal/mol-kelvin<br>Entropy (S) = 189.629 cal/mol-kelvin
**4**	Electronic Energy (EE) = -1052.5493 Hartree<br>Zero-point Energy Correction = 0.333979 Hartree<br>Thermal Correction to Energy = 0.358543 Hartree<br>Thermal Correction to Enthalpy = 0.359487 Hartree<br>Thermal Correction to Free Energy = 0.276935 Hartree<br>EE + Zero-point Energy = -1052.2153 Hartree<br>EE + Thermal Energy Correction = -1052.1908 Hartree<br>EE + Thermal Enthalpy Correction = -1052.1898 Hartree<br>EE + Thermal Free Energy Correction = -1052.2724 Hartree<br>E (Thermal) = 224.989 kcal/mol<br>Heat Capacity (Cv) = 88.473 cal/mol-kelvin<br>Entropy (S) = 173.745 cal/mol-kelvin

Table S4: Thermochemical parameters and energy corrections obtained from DFT calculations, including electronic energy, zero-point energy, thermal corrections, and thermodynamic properties (E, Cv, S) at standard conditions.

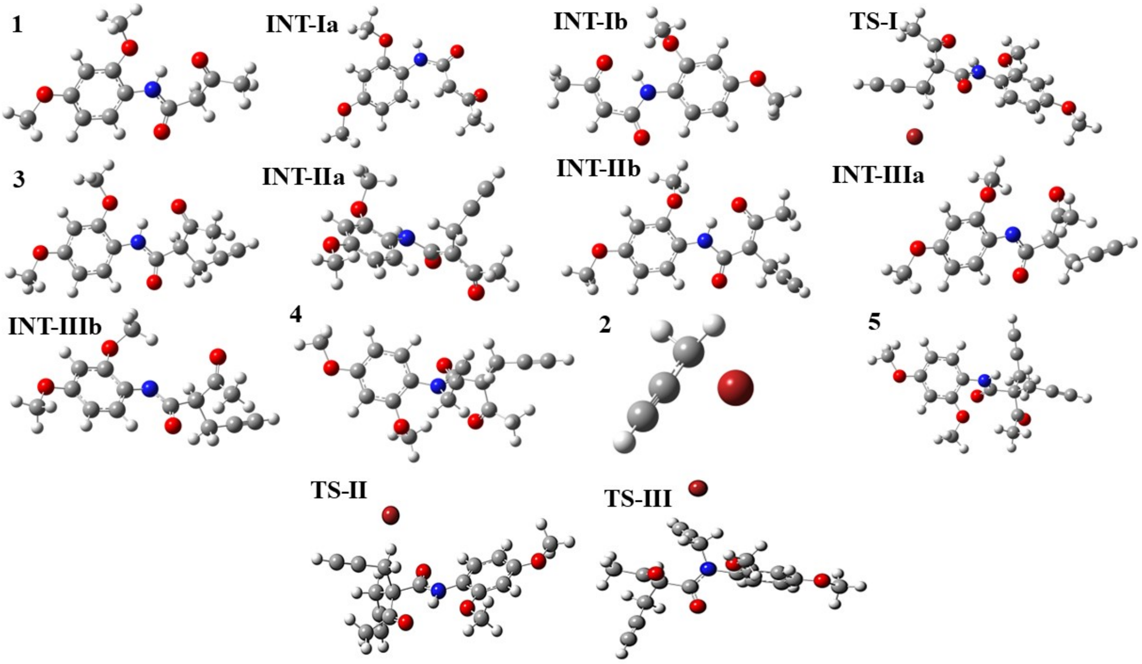

Figure S29: Optimized structure of all molecules

1

Input orientation:

Center Number	Atomic Number	Atomic Type	Coordinates (Angstroms) X	Y	Z
1	6	0	-3.123276	-0.105714	0.094633
2	6	0	-2.347666	1.060795	-0.010837
3	6	0	-0.967940	0.953627	-0.123151
4	6	0	-0.328400	-0.311956	-0.131402
5	6	0	-1.118358	-1.459963	-0.025733
6	6	0	-2.514437	-1.361548	0.086633
7	7	0	1.080496	-0.299293	-0.251549
8	6	0	1.915127	-1.376049	-0.286889
9	6	0	3.405473	-1.068350	-0.508031
10	6	0	3.984279	0.167553	0.160455
11	6	0	5.354294	0.037882	0.777575
12	8	0	3.362610	1.247603	0.181898
13	8	0	1.553095	-2.573890	-0.208675
14	8	0	-4.496060	0.111645	0.202289
15	6	0	-5.369779	-1.040496	0.315415
16	8	0	-0.102663	2.038433	-0.237664
17	6	0	-0.644658	3.383127	-0.196012
18	1	0	-2.854698	2.016002	-0.002640
19	1	0	-0.634221	-2.425388	-0.030586
20	1	0	-3.097014	-2.270148	0.167310
21	1	0	1.510779	0.626016	-0.263799
22	1	0	3.951287	-1.966290	-0.211838
23	1	0	3.557538	-0.949030	-1.593142

	24 1 0 6.069036 -0.375348 0.054929<br>25 1 0 5.702443 1.011724 1.125750<br>26 1 0 5.317844 -0.659764 1.624741<br>27 1 0 -6.376838 -0.630485 0.385351<br>28 1 0 -5.293357 -1.686473 -0.567396<br>29 1 0 -5.144259 -1.624418 1.215776<br>30 1 0 0.219144 4.041003 -0.280007<br>31 1 0 -1.330360 3.556467 -1.033246<br>32 1 0 -1.164616 3.567415 0.750916<br>---
2	Input orientation:<br>---<br>Center Atomic Atomic Coordinates (Angstroms)<br>Number Number Type X Y Z<br>---<br>1 6 0 2.859328 -0.515662 -0.000082<br>2 6 0 1.837327 0.138365 0.000126<br>3 6 0 0.660684 0.964670 -0.000025<br>4 35 0 -1.057475 -0.158983 -0.000000<br>5 1 0 3.742228 -1.111764 0.000077<br>6 1 0 0.562636 1.576064 0.895757<br>7 1 0 0.562730 1.575884 -0.895940
3	Input orientation:<br>---<br>Center Atomic Atomic Coordinates (Angstroms)<br>Number Number Type X Y Z<br>---<br>1 6 0 -3.841521 -0.379704 0.022024<br>2 6 0 -3.244554 0.880212 -0.161482<br>3 6 0 -1.860357 0.977012 -0.227731<br>4 6 0 -1.041457 -0.176198 -0.112689<br>5 6 0 -1.654276 -1.418993 0.067448<br>6 6 0 -3.052539 -1.526947 0.135566<br>7 7 0 0.354377 0.045326 -0.194542<br>8 6 0 1.356401 -0.872002 -0.129324<br>9 6 0 2.781554 -0.279926 -0.312782<br>10 6 0 3.006698 0.875731 0.673525<br>11 6 0 3.881775 0.682293 1.879590<br>12 8 0 2.412438 1.955361 0.465680<br>13 8 0 1.187168 -2.107979 0.024102<br>14 6 0 3.836283 -1.407976 -0.270488<br>15 6 0 5.166412 -0.965891 -0.698741<br>16 6 0 6.265010 -0.594900 -1.059596<br>17 8 0 -1.163153 2.166706 -0.406531<br>18 6 0 -1.908010 3.415611 -0.533931<br>19 8 0 -5.232407 -0.369067 0.076215<br>20 6 0 -5.931702 -1.634473 0.271151<br>21 1 0 -3.883563 1.748623 -0.246791<br>22 1 0 -1.036444 -2.300250 0.154080<br>23 1 0 -3.496011 -2.503711 0.276278

	24	1	0	0.621567	1.023536	-0.287073
	25	1	0	2.799152	0.179963	-1.310180
	26	1	0	4.907300	0.437191	1.582045
	27	1	0	3.884344	1.594602	2.477686
	28	1	0	3.514427	-0.151428	2.491355
	29	1	0	3.882880	-1.841457	0.735586
	30	1	0	3.482772	-2.217079	-0.918802
	31	1	0	7.229743	-0.272876	-1.377943
	32	1	0	-1.150873	4.187027	-0.661610
	33	1	0	-2.565306	3.386287	-1.408248
	34	1	0	-2.494127	3.610649	0.369280
	35	1	0	-6.989871	-1.377547	0.281016
	36	1	0	-5.725285	-2.328697	-0.550070
	37	1	0	-5.652005	-2.095371	1.224218
4	Input orientation:					

	Center	Atomic	Atomic	Coordinates (Angstroms)		
	Number	Number	Type	X	Y	Z

	1	6	0	4.025609	-0.204404	-0.258772
	2	6	0	3.411491	1.002288	0.108456
	3	6	0	2.033637	1.049059	0.303432
	4	6	0	1.246814	-0.110025	0.146487
	5	6	0	1.874817	-1.306643	-0.211942
	6	6	0	3.255835	-1.365345	-0.424830
	7	7	0	-0.171240	-0.061092	0.400364
	8	6	0	-1.058550	-0.318419	-0.622375
	9	6	0	-2.556492	-0.340481	-0.282788
	10	6	0	-3.104021	1.093711	-0.160482
	11	6	0	-4.274405	1.323868	0.756412
	12	8	0	-2.595102	2.016197	-0.826228
	13	8	0	-0.665975	-0.529141	-1.794945
	14	6	0	-0.585711	0.172617	1.810197
	15	6	0	-3.308501	-1.108961	-1.423662
	16	6	0	-4.711177	-1.408022	-1.134952
	17	6	0	-0.736455	-1.068783	2.578738
	18	6	0	-0.863776	-2.098411	3.208581
	19	6	0	-5.873022	-1.680653	-0.909642
	20	8	0	1.452655	2.251712	0.733619
	21	6	0	0.809165	3.075067	-0.311510
	22	8	0	5.400914	-0.146580	-0.432570
	23	6	0	6.113689	-1.361334	-0.819923
	24	1	0	4.009094	1.895219	0.242549
	25	1	0	1.275746	-2.202071	-0.328611
	26	1	0	3.710608	-2.306773	-0.703006
	27	1	0	-2.728978	-0.876264	0.655771
	28	1	0	-4.006567	1.079263	1.792217
	29	1	0	-4.593014	2.366075	0.701725
	30	1	0	-5.105717	0.664378	0.482517
	31	1	0	0.171696	0.821713	2.256280

	32	1	0	-1.512508	0.751342	1.830010
	33	1	0	-3.215731	-0.529895	-2.349121
	34	1	0	-2.762413	-2.043059	-1.598525
	35	1	0	-0.972668	-3.003560	3.761066
	36	1	0	-6.893040	-1.921009	-0.715379
	37	1	0	0.488527	3.983523	0.197513
	38	1	0	1.533837	3.318426	-1.094425
	39	1	0	-0.054738	2.559466	-0.737659
	40	1	0	7.158804	-1.065540	-0.894215
	41	1	0	6.000738	-2.141475	-0.060382
	42	1	0	5.761384	-1.730385	-1.788439
INT-Ia	Input orientation:					

	Center Number	Atomic Number	Atomic Type	Coordinates (Angstroms) X	Y	Z

	1	6	0	-2.764897	-0.884037	-0.051898
	2	6	0	-2.704367	0.500726	0.183202
	3	6	0	-1.484399	1.160737	0.095787
	4	6	0	-0.282694	0.467304	-0.228889
	5	6	0	-0.383857	-0.905734	-0.488129
	6	6	0	-1.609077	-1.587809	-0.389909
	7	7	0	0.886127	1.236218	-0.331681
	8	6	0	2.271840	0.911683	-0.313460
	9	6	0	2.666542	-0.348755	0.216931
	10	6	0	3.969063	-0.898393	0.229297
	11	6	0	4.126555	-2.245282	0.951769
	12	8	0	5.034318	-0.412922	-0.302979
	13	8	0	3.032231	1.850137	-0.739027
	14	1	0	1.896874	-0.933272	0.704404
	15	8	0	-1.325729	2.533000	0.294983
	16	6	0	-2.502840	3.341850	0.583840
	17	8	0	-4.036458	-1.452691	0.074669
	18	6	0	-4.177199	-2.881478	-0.169345
	19	1	0	-3.619435	1.021568	0.432314
	20	1	0	0.501419	-1.454181	-0.779965
	21	1	0	-1.634241	-2.650958	-0.592148
	22	1	0	0.727853	2.227852	-0.459516
	23	1	0	3.194786	-2.614165	1.391156
	24	1	0	4.506544	-2.999059	0.250002
	25	1	0	4.875468	-2.147661	1.748413
	26	1	0	-2.133709	4.361737	0.680337
	27	1	0	-3.228845	3.283002	-0.233739
	28	1	0	-2.973764	3.026279	1.520559
	29	1	0	-5.233378	-3.098248	-0.013886
	30	1	0	-3.893586	-3.137316	-1.196175
	31	1	0	-3.571795	-3.464163	0.533672
INT-Ib	Input orientation:					

	Center	Atomic	Atomic	Coordinates (Angstroms)		

	Number	Number	Type	X	Y	Z
	1	6	0	3.161514	0.064344	-0.091021
	2	6	0	2.313497	1.172261	-0.230651
	3	6	0	0.934663	0.997682	-0.221183
	4	6	0	0.342488	-0.284496	-0.074364
	5	6	0	1.216060	-1.381210	0.065681
	6	6	0	2.608023	-1.211165	0.060590
	7	7	0	-1.053042	-0.361980	-0.088264
	8	6	0	-1.865358	-1.484317	0.096719
	9	6	0	-3.282604	-1.237823	0.063153
	10	6	0	-3.895534	0.006403	-0.116631
	11	6	0	-5.417347	0.076872	-0.109133
	12	8	0	-1.369114	-2.651751	0.286651
	13	8	0	-3.262064	1.137281	-0.294718
	14	1	0	-3.901270	-2.116859	0.201623
	15	8	0	4.533269	0.341975	-0.115334
	16	6	0	5.462257	-0.770744	0.021280
	17	8	0	0.102250	2.122822	-0.407053
	18	6	0	-0.308534	2.816485	0.826674
	19	1	0	2.732593	2.164062	-0.353330
	20	1	0	0.781065	-2.362546	0.182535
	21	1	0	3.238895	-2.084651	0.171675
	22	1	0	-1.592737	0.503903	-0.251272
	23	1	0	-5.776149	0.494183	-1.058842
	24	1	0	-5.884225	-0.900711	0.043344
	25	1	0	-5.755619	0.756069	0.684054
	26	1	0	6.455325	-0.325235	-0.025854
	27	1	0	5.330997	-1.281204	0.981865
	28	1	0	5.340713	-1.491174	-0.795218
	29	1	0	-0.858677	3.698443	0.499168
	30	1	0	-0.960069	2.176898	1.428715
	31	1	0	0.570979	3.114314	1.406330
TS-I	Input orientation:					
	Center Number	Atomic Number	Atomic Type	Coordinates (Angstroms) X	Y	Z
	1	6	0	4.479580	-0.525344	0.147357
	2	6	0	3.964840	0.370897	-0.801399
	3	6	0	2.688174	0.891824	-0.649231
	4	6	0	1.880419	0.531660	0.463503
	5	6	0	2.417105	-0.360164	1.391316
	6	6	0	3.706144	-0.890480	1.243165
	7	7	0	0.600895	1.103724	0.530900
	8	6	0	-0.359678	0.956291	1.505402
	9	6	0	-1.615329	1.667149	1.282811
	10	6	0	-1.861897	2.635047	0.269431
	11	6	0	-3.125719	3.470714	0.377207
	12	8	0	-1.101832	2.840720	-0.713430

	13	8	0	-0.192645	0.237447	2.511579
	14	6	0	-2.760658	-0.331422	0.220649
	15	6	0	-4.095698	0.057164	0.459284
	16	6	0	-5.239336	0.382266	0.667397
	17	8	0	2.113781	1.766956	-1.525653
	18	6	0	2.853439	2.177913	-2.676428
	19	8	0	5.749445	-0.976705	-0.103498
	20	6	0	6.332852	-1.892664	0.822418
	21	1	0	4.584818	0.639381	-1.645351
	22	1	0	1.815290	-0.641553	2.240902
	23	1	0	4.075326	-1.578740	1.990707
	24	1	0	0.331496	1.738644	-0.225211
	25	1	0	-2.282813	1.642753	2.133641
	26	1	0	-2.844423	4.510526	0.574055
	27	1	0	-3.660223	3.452677	-0.576374
	28	1	0	-3.792724	3.133223	1.170332
	29	1	0	-2.192230	-0.793341	1.009398
	30	1	0	-6.247485	0.671770	0.848043
	31	1	0	2.202571	2.861473	-3.217641
	32	1	0	3.771951	2.697416	-2.388834
	33	1	0	3.097093	1.322847	-3.313308
	34	1	0	7.325189	-2.116511	0.435250
	35	1	0	6.422406	-1.447283	1.817975
	36	1	0	5.750238	-2.816733	0.886725
	37	1	0	-2.242174	0.024127	-0.652667
	38	35	0	-3.052424	-2.539657	-0.860485
INT-IIa	Input orientation:					

	Center	Atomic	Atomic	Coordinates (Angstroms)		
	Number	Number	Type	X	Y	Z

	1	6	0	-3.282112	-0.744515	0.041684
	2	6	0	-3.181829	0.646633	-0.112473
	3	6	0	-1.947305	1.224799	-0.378357
	4	6	0	-0.768135	0.446801	-0.501709
	5	6	0	-0.896752	-0.944879	-0.360184
	6	6	0	-2.136603	-1.538205	-0.080960
	7	7	0	0.423493	1.121113	-0.814668
	8	6	0	1.732390	0.601393	-1.091627
	9	6	0	2.305271	-0.361279	-0.196163
	10	6	0	3.404931	-1.186520	-0.583085
	11	6	0	4.035047	-2.094859	0.484927
	12	8	0	3.899785	-1.278460	-1.763602
	13	8	0	2.292775	1.121636	-2.113760
	14	6	0	1.838125	-0.388685	1.265502
	15	6	0	2.654210	0.455365	2.161978
	16	6	0	3.331151	1.159119	2.888354
	17	8	0	-4.563963	-1.233343	0.312273
	18	6	0	-4.734628	-2.671667	0.465902
	19	8	0	-1.855466	2.617597	-0.590310

	20	6	0	-1.580106	3.417298	0.617939
	21	1	0	-4.067221	1.266016	-0.032044
	22	1	0	-0.019094	-1.568895	-0.476479
	23	1	0	-2.187446	-2.614764	0.023547
	24	1	0	0.268719	2.013100	-1.272037
	25	1	0	3.344143	-2.888176	0.802384
	26	1	0	4.920180	-2.565705	0.050318
	27	1	0	4.327996	-1.540354	1.383796
	28	1	0	0.799968	-0.054583	1.342990
	29	1	0	1.836620	-1.413739	1.659692
	30	1	0	3.925835	1.778940	3.518527
	31	1	0	-5.795920	-2.816264	0.664268
	32	1	0	-4.453110	-3.203753	-0.449460
	33	1	0	-4.145494	-3.052562	1.307408
	34	1	0	-1.534585	4.453034	0.282109
	35	1	0	-2.386867	3.292529	1.346773
	36	1	0	-0.625151	3.123163	1.063705
INT-IIb	Input orientation:					
	--					
	Center Number	Atomic Number	Atomic Type	Coordinates (Angstroms) X	Y	Z
	--					
	1	6	0	-3.804846	-0.361309	0.144321
	2	6	0	-3.202413	0.877115	0.407170
	3	6	0	-1.820767	1.007944	0.327629
	4	6	0	-0.981765	-0.085631	-0.013497
	5	6	0	-1.611844	-1.320221	-0.268006
	6	6	0	-3.005300	-1.458003	-0.193734
	7	7	0	0.395563	0.149855	-0.066519
	8	6	0	1.415102	-0.746382	-0.382512
	9	6	0	2.763433	-0.215140	-0.390989
	10	6	0	3.080080	1.127346	-0.110833
	11	6	0	4.538530	1.576742	-0.160879
	12	8	0	2.205804	2.050854	0.189549
	13	8	0	1.156117	-1.974227	-0.660525
	14	6	0	3.854871	-1.224817	-0.729431
	15	6	0	4.580529	-1.766079	0.436259
	16	6	0	5.170752	-2.203002	1.406785
	17	8	0	-5.200164	-0.392837	0.250803
	18	6	0	-5.880270	-1.655905	0.001492
	19	8	0	-1.215980	2.244735	0.636965
	20	6	0	-1.282201	3.268354	-0.418015
	21	1	0	-3.815847	1.727370	0.681890
	22	1	0	-0.988520	-2.161884	-0.529572
	23	1	0	-3.443489	-2.426731	-0.400713
	24	1	0	0.738690	1.100219	0.150267
	25	1	0	5.183385	0.949866	0.465707
	26	1	0	4.938664	1.532780	-1.183160
	27	1	0	4.597215	2.610742	0.187572
	28	1	0	3.372153	-2.058239	-1.253618

	29	1	0	4.587866	-0.795049	-1.426532
	30	1	0	5.681912	-2.585651	2.259519
	31	1	0	-6.939907	-1.446119	0.142986
	32	1	0	-5.705798	-2.004806	-1.022368
	33	1	0	-5.557599	-2.426468	0.710398
	34	1	0	-0.805791	4.154629	0.000379
	35	1	0	-0.738801	2.937566	-1.308710
	36	1	0	-2.322630	3.488507	-0.677299
**TS-II**	Input orientation:					
	Center Number	Atomic Number	Atomic Type	Coordinates (Angstroms) X	Y	Z
	1	6	0	-4.360126	-0.624412	-0.123179
	2	6	0	-3.845386	0.271829	0.825577
	3	6	0	-2.568720	0.792757	0.673410
	4	6	0	-1.760965	0.432593	-0.439325
	5	6	0	-2.297651	-0.459231	-1.367138
	6	6	0	-3.586690	-0.989548	-1.218987
	7	7	0	-0.481441	1.004656	-0.506722
	8	6	0	0.479132	0.857224	-1.481224
	9	6	0	1.734783	1.568082	-1.258633
	10	6	0	1.981351	2.535980	-0.245253
	11	6	0	3.245173	3.371647	-0.353028
	12	8	0	1.221286	2.741653	0.737609
	13	8	0	0.312099	0.138380	-2.487401
	14	6	0	2.880112	-0.430490	-0.196471
	15	6	0	4.215152	-0.041903	-0.435106
	16	6	0	5.358790	0.283199	-0.643219
	17	8	0	-1.994327	1.667889	1.549831
	18	6	0	-2.733985	2.078846	2.700606
	19	8	0	-5.629991	-1.075773	0.127676
	20	6	0	-6.213398	-1.991732	-0.798240
	21	1	0	-4.465364	0.540313	1.669529
	22	1	0	-1.695836	-0.740621	-2.216724
	23	1	0	-3.955872	-1.677807	-1.966529
	24	1	0	-0.212042	1.639576	0.249389
	25	1	0	2.963877	4.411459	-0.549877
	26	1	0	3.779677	3.353609	0.600552
	27	1	0	3.912178	3.034156	-1.146154
	28	1	0	2.311684	-0.892408	-0.985220
	29	1	0	6.366938	0.572703	-0.823865
	30	1	0	-2.083117	2.762405	3.241819
	31	1	0	-3.652497	2.598348	2.413012
	32	1	0	-2.977639	1.223779	3.337486
	33	1	0	-7.205735	-2.215578	-0.411072
	34	1	0	-6.302953	-1.546350	-1.793797
	35	1	0	-5.630784	-2.915801	-0.862547
	36	1	0	2.361628	-0.074941	0.676845
	37	35	0	3.171878	-2.638725	0.884663

	38	6	0	2.685084	1.533349	-2.469964
	39	1	0	3.663081	1.842260	-2.165013
	40	1	0	2.730695	0.537651	-2.859072
	41	6	0	2.162153	2.487075	-3.560170
	42	6	0	1.754267	3.230982	-4.410531
	43	1	0	1.390931	3.893636	-5.168012
**5**	Input orientation:					

	Center Number	Atomic Number	Atomic Type	Coordinates (Angstroms) X	Y	Z

	1	6	0	2.142412	-1.514639	0.311574
	2	1	0	1.641397	-2.474147	0.379823
	3	6	0	3.537984	-1.462806	0.199344
	4	1	0	4.109073	-2.381119	0.186490
	5	6	0	4.156587	-0.211375	0.097848
	6	6	0	3.397779	0.971083	0.118063
	7	1	0	3.921141	1.914287	0.042507
	8	6	0	2.010505	0.904119	0.243465
	9	6	0	1.370778	-0.354701	0.334797
	10	6	0	-0.924531	-0.142374	-0.514465
	11	6	0	-2.440283	-0.170344	-0.213564
	12	6	0	-2.787428	-0.283933	1.307465
	13	1	0	-2.443286	-1.262842	1.667414
	14	1	0	-2.239400	0.481090	1.871001
	15	6	0	-4.220924	-0.154311	1.579346
	16	6	0	-5.409056	-0.041736	1.800233
	17	1	0	-6.452309	0.057000	1.994953
	18	6	0	-3.066636	-1.353818	-1.015683
	19	1	0	-4.156425	-1.290635	-0.919960
	20	1	0	-2.837634	-1.196128	-2.074766
	21	6	0	-2.589604	-2.667632	-0.580311
	22	6	0	-2.192140	-3.755656	-0.215066
	23	1	0	-1.845806	-4.713637	0.098218
	24	6	0	-2.999160	1.169084	-0.765941
	25	6	0	-2.494153	2.446934	-0.141742
	26	1	0	-2.732131	3.289836	-0.793398
	27	1	0	-1.416892	2.413403	0.046924
	28	1	0	-2.997142	2.608925	0.820812
	29	6	0	1.795987	3.345131	0.199993
	30	1	0	2.490343	3.517104	1.028137
	31	1	0	0.962285	4.041913	0.262395
	32	1	0	2.314816	3.467192	-0.755592
	33	6	0	6.396071	-1.197098	-0.057081
	34	1	0	6.311306	-1.773342	0.869795
	35	1	0	7.405130	-0.800163	-0.155513
	36	1	0	6.161642	-1.835626	-0.914779
	37	7	0	-0.052507	-0.429922	0.489473
	38	1	0	-0.403836	-0.700391	1.396851
	39	8	0	5.526601	-0.024358	-0.025345

	40 8 0 1.189414 2.018329 0.296546<br>41 8 0 -0.553775 0.142825 -1.680669<br>42 8 0 -3.853471 1.174599 -1.666441
**INT-IIIa**	Input orientation:<br>---<br>Center Atomic Atomic Coordinates (Angstroms)<br>Number Number Type X Y Z<br>---<br>1 6 0 -3.872255 -0.218099 0.119016<br>2 6 0 -3.212715 1.016425 0.190482<br>3 6 0 -1.824990 1.085353 0.091401<br>4 6 0 -1.024539 -0.084026 -0.085886<br>5 6 0 -1.730578 -1.309220 -0.172529<br>6 6 0 -3.127095 -1.385533 -0.070691<br>7 7 0 0.369308 0.080697 -0.136978<br>8 6 0 1.223652 -0.926723 -0.333599<br>9 6 0 2.706689 -0.459117 -0.410136<br>10 6 0 2.872425 0.939884 0.152582<br>11 6 0 2.829327 1.124450 1.653331<br>12 8 0 3.096411 1.911852 -0.602755<br>13 8 0 1.003764 -2.186758 -0.494785<br>14 6 0 3.627620 -1.511992 0.265439<br>15 6 0 5.057264 -1.270985 0.058827<br>16 6 0 6.240118 -1.056473 -0.120840<br>17 8 0 -1.265349 2.367765 0.241662<br>18 6 0 -0.503039 2.898173 -0.897522<br>19 8 0 -5.268512 -0.170335 0.238000<br>20 6 0 -6.006000 -1.421758 0.153999<br>21 1 0 -3.782752 1.927865 0.327170<br>22 1 0 -1.147312 -2.208309 -0.312714<br>23 1 0 -3.608021 -2.354584 -0.136522<br>24 1 0 2.975934 -0.401541 -1.473739<br>25 1 0 3.803570 0.859402 2.086633<br>26 1 0 2.627221 2.171692 1.889115<br>27 1 0 2.068291 0.489517 2.111713<br>28 1 0 3.407001 -1.559546 1.340347<br>29 1 0 3.326782 -2.483139 -0.145138<br>30 1 0 7.277778 -0.872854 -0.278266<br>31 1 0 -0.231090 3.916450 -0.613816<br>32 1 0 0.386389 2.291625 -1.067761<br>33 1 0 -1.132072 2.922616 -1.795718<br>34 1 0 -7.054719 -1.148641 0.267781<br>35 1 0 -5.853784 -1.908200 -0.816117<br>36 1 0 -5.714207 -2.109577 0.955654
**INT-IIIb**	Input orientation:<br>---<br>Center Atomic Atomic Coordinates (Angstroms)<br>Number Number Type X Y Z<br>---<br>1 6 0 -3.942972 -0.260963 -0.080910

	2	6	0	-3.380932	0.992764	-0.359902
	3	6	0	-1.998741	1.147347	-0.430374
	4	6	0	-1.107447	0.051642	-0.221457
	5	6	0	-1.713578	-1.193173	0.076891
	6	6	0	-3.104628	-1.356996	0.144382
	7	7	0	0.266678	0.303246	-0.359464
	8	6	0	1.206433	-0.609863	-0.119013
	9	6	0	2.654514	-0.062894	-0.339683
	10	6	0	3.191299	0.418888	1.000786
	11	6	0	3.570161	-0.622779	2.025660
	12	8	0	3.272291	1.641013	1.254460
	13	8	0	1.101632	-1.835082	0.282860
	14	6	0	3.537442	-1.158623	-0.995800
	15	6	0	4.942226	-0.774446	-1.158402
	16	6	0	6.104266	-0.441377	-1.285181
	17	8	0	-1.538715	2.430557	-0.782034
	18	6	0	-0.716547	3.134569	0.211496
	19	8	0	-5.343610	-0.304709	-0.042014
	20	6	0	-5.981139	-1.577595	0.259544
	21	1	0	-4.023636	1.849098	-0.527326
	22	1	0	-1.059452	-2.036767	0.247800
	23	1	0	-3.509874	-2.337059	0.367596
	24	1	0	2.585965	0.811251	-0.989323
	25	1	0	4.535589	-1.072909	1.759584
	26	1	0	3.660699	-0.164235	3.012689
	27	1	0	2.826342	-1.426103	2.034250
	28	1	0	3.447292	-2.073289	-0.398917
	29	1	0	3.103099	-1.396320	-1.976050
	30	1	0	7.123894	-0.153552	-1.398285
	31	1	0	-0.507879	4.112672	-0.225416
	32	1	0	-1.275587	3.260860	1.146573
	33	1	0	0.207852	2.584117	0.390159
	34	1	0	-7.052039	-1.377395	0.240125
	35	1	0	-5.732908	-2.334372	-0.493090
	36	1	0	-5.691035	-1.941950	1.251486
**TS-III**	Input orientation:					

	Center Number	Atomic Number	Atomic Type	Coordinates (Angstroms) X	Y	Z

	1	6	0	4.075815	-0.973464	-0.118510
	2	6	0	3.308027	-0.623764	1.001190
	3	6	0	1.924330	-0.503180	0.898437
	4	6	0	1.265417	-0.736048	-0.338650
	5	6	0	2.065977	-1.070499	-1.430203
	6	6	0	3.457432	-1.199049	-1.345149
	7	7	0	-0.118967	-0.542429	-0.497520
	8	6	0	-0.961427	-1.531307	-0.215942
	9	6	0	-2.442112	-1.195472	-0.476138
	10	6	0	-3.202383	-0.767284	0.781333

	11	6	0	-4.669088	-0.430243	0.610966
	12	8	0	-2.661164	-0.670410	1.866932
	13	8	0	-0.664125	-2.685767	0.175119
	14	6	0	-0.556048	1.788300	-0.509060
	15	6	0	-1.073976	1.836541	-1.826592
	16	6	0	-1.515639	1.888566	-2.947497
	17	8	0	1.125064	-0.154068	1.948195
	18	6	0	1.729723	0.090857	3.216798
	19	8	0	5.426617	-1.062075	0.103162
	20	6	0	6.267743	-1.410265	-0.995507
	21	1	0	3.823025	-0.452308	1.936422
	22	1	0	1.576548	-1.238165	-2.383464
	23	1	0	4.023812	-1.466296	-2.226479
	24	1	0	-5.245897	-1.355868	0.518901
	25	1	0	-5.023671	0.117125	1.483717
	26	1	0	-4.841905	0.154454	-0.295670
	27	1	0	0.507453	1.756902	-0.357590
	28	1	0	-1.905354	1.932572	-3.936861
	29	1	0	0.913740	0.348865	3.889290
	30	1	0	2.241365	-0.800906	3.591767
	31	1	0	2.437063	0.924455	3.166845
	32	1	0	7.282407	-1.424718	-0.601622
	33	1	0	6.016620	-2.399614	-1.390069
	34	1	0	6.199629	-0.669363	-1.798056
	35	1	0	-1.191266	1.522871	0.315339
	36	35	0	-0.621467	4.117026	0.123605
	37	6	0	-3.177138	-2.332691	-1.247661
	38	1	0	-4.062861	-1.913549	-1.734757
	39	1	0	-2.519180	-2.670944	-2.052811
	40	6	0	-3.598771	-3.485802	-0.454193
	41	6	0	-4.004286	-4.428066	0.176846
	42	1	0	-4.344119	-5.263701	0.740947
	43	1	0	-2.476003	-0.324935	-1.139452
**4**	Input orientation:					
	Center Number	Atomic Number	Atomic Type	Coordinates (Angstroms) X	Y	Z
	1	6	0	4.025609	-0.204404	-0.258772
	2	6	0	3.411491	1.002288	0.108456
	3	6	0	2.033637	1.049059	0.303432
	4	6	0	1.246814	-0.110025	0.146487
	5	6	0	1.874817	-1.306643	-0.211942
	6	6	0	3.255835	-1.365345	-0.424830
	7	7	0	-0.171240	-0.061092	0.400364
	8	6	0	-1.058550	-0.318419	-0.622375
	9	6	0	-2.556492	-0.340481	-0.282788
	10	6	0	-3.104021	1.093711	-0.160482
	11	6	0	-4.274405	1.323868	0.756412
	12	8	0	-2.595102	2.016197	-0.826228

	13	8	0	-0.665975	-0.529141	-1.794945
	14	6	0	-0.585711	0.172617	1.810197
	15	6	0	-3.308501	-1.108961	-1.423662
	16	6	0	-4.711177	-1.408022	-1.134952
	17	6	0	-0.736455	-1.068783	2.578738
	18	6	0	-0.863776	-2.098411	3.208581
	19	6	0	-5.873022	-1.680653	-0.909642
	20	8	0	1.452655	2.251712	0.733619
	21	6	0	0.809165	3.075067	-0.311510
	22	8	0	5.400914	-0.146580	-0.432570
	23	6	0	6.113689	-1.361334	-0.819923
	24	1	0	4.009094	1.895219	0.242549
	25	1	0	1.275746	-2.202071	-0.328611
	26	1	0	3.710608	-2.306773	-0.703006
	27	1	0	-2.728978	-0.876264	0.655771
	28	1	0	-4.006567	1.079263	1.792217
	29	1	0	-4.593014	2.366075	0.701725
	30	1	0	-5.105717	0.664378	0.482517
	31	1	0	0.171696	0.821713	2.256280
	32	1	0	-1.512508	0.751342	1.830010
	33	1	0	-3.215731	-0.529895	-2.349121
	34	1	0	-2.762413	-2.043059	-1.598525
	35	1	0	-0.972668	-3.003560	3.761066
	36	1	0	-6.893040	-1.921009	-0.715379
	37	1	0	0.488527	3.983523	0.197513
	38	1	0	1.533837	3.318426	-1.094425
	39	1	0	-0.054738	2.559466	-0.737659
	40	1	0	7.158804	-1.065540	-0.894215
	41	1	0	6.000738	-2.141475	-0.060382
	42	1	0	5.761384	-1.730385	-1.788439
